# Rational Design of Conjugated Phenylpropanoid–Polyene Hybrids: Density Functional Theory Insights into Antiradical and Optical Properties

**DOI:** 10.3390/ijms27083378

**Published:** 2026-04-09

**Authors:** Marcin Molski

**Affiliations:** Department of Quantum Chemistry, Faculty of Chemistry, Adam Mickiewicz University of Poznań, ul. Uniwersytetu Poznańskiego 8, 61-614 Poznań, Poland; mamolski@amu.edu.pl; Tel.: +48-602-250-063

**Keywords:** phenylpropanoids, polyenoic acids, polyenals, radical scavengers, thermodynamic descriptors, chemical activity descriptors

## Abstract

A structural analysis of phenylpropanoids demonstrates that the benzene ring and the propenoic fragment act as two largely independent π-electron systems. This distinctive feature provides a theoretical basis for the rational design of novel compounds obtained through the structural integration of phenylpropanoids with polyene aldehydes and acids. These classes may be combined by elongating the carbon backbone via iterative vinyl group extension, thereby generating an expanded conjugated double-bond system. Alternatively, the structure of polyene aldehydes may be modified by replacing the unreactive methyl group with a benzene ring bearing suitable functional substituents. DFT computational studies performed at the B3LYP/QZVP level of theory indicate that the designed analogs predominantly scavenge radicals through the sequential proton loss electron transfer (SPLET) mechanism in aqueous environments. This pathway involves the initial deprotonation of carboxyl, aldehyde, or phenolic groups, with the hydroxyl moiety exhibiting the greatest propensity for proton dissociation. Carbon chain extension exerts only a minor influence on proton affinity (PA) values but significantly affects electron transfer enthalpy (ETE) parameters. Consequently, increasing the number of conjugated double bonds enhances activation of the second step of the SPLET mechanism, thereby improving overall radical-scavenging activity. Comparison of the calculated chemical reactivity parameters substantiates the conclusions drawn from the thermodynamic analysis. A pronounced enhancement in the reactivity of the modeled compounds, relative to the parent constituents, is observed. Time-dependent density functional theory (TD-DFT) calculations further predict absorption in the visible region, indicating potential applications of the modeled compounds as radical-scavenging dyes in food, pharmaceutical, cosmetic, and dietary supplement formulations.

## 1. Introduction

**Figure 1:**
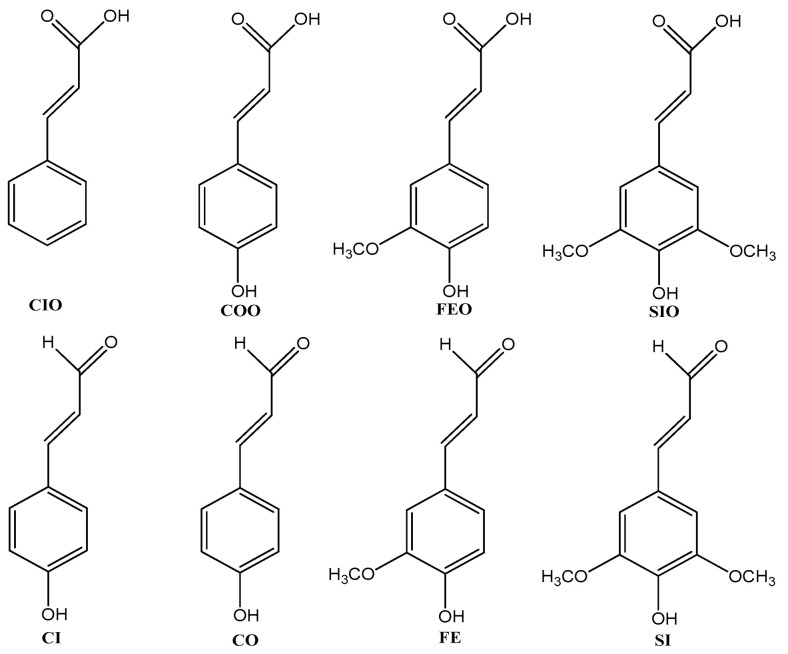
The trans-phenylpropanoid acids: cinnamic (CIO), coumaric (COO), ferulic (FEO), sinapic (SIO), and their aldehyde forms (CI, CO, FE, SI) considered in this work.

Throughout evolution, organisms have developed complex defense mechanisms that protect against the deleterious effects of free radicals [1,2,3]. These mechanisms comprise an endogenous antioxidant defense system, including both enzymatic and non-enzymatic components, which function to neutralize reactive oxygen species (ROS) and reactive nitrogen species (RNS) generated predominantly during metabolic processes. This system is further reinforced by naturally occurring antioxidants present in food, as well as those provided through dietary supplementation. One notable class of non-enzymatic scavengers comprises conjugated polyenes, which are found in plants, fungi, and animals [4]. They are responsible for their coloring and provides protection against microorganisms, photoinhibition, UV radiation, and radical damage to DNA, RNA, lipids, cells, and tissues [5,6,7,8,9,10]. The class of conjugated polyenes includes [4]: tetraterpenes (carotenoids), unmethylated linear polyenals (polyunsaturated aldehydes), and laetiporic acids (isolated from fungus *Laetiporus sulphureus*). They have been identified in parrot feather pigments (6–9 conjugated double bonds in the carbon chain) [11,12,13,14], pearls, mollusk shells, and octocorals (6–14 double bonds) [15,16,17,18,19,20,21,22,23,24]. The radical scavengers identified in plants (vitamins A, E, C, phenolic and polyphenolic compounds, flavonoids, terpenoids) are produced in a chain of metabolic reactions and play an important role in the plant’s protection against pests, insects, diseases, and wounds. The consumption of plants and the use of supplements rich in radical-scavenging substances enhance the endogenous enzyme-based antiradical defense system, which becomes inefficient with age and certain disease states [25]. Among the most common and potent anti-radical compounds widespread in the plant kingdom are phenylpropanoid acids and their aldehyde forms, of which cinnamic acid (CIO, trans-3-phenylprop-2-enoic acid) and cinnamaldehyde (CI, trans-3-phenylprop-2-enal), as well as their derivatives (see Figure 1), deserve special attention due to their broad spectrum of bioactivity [3,25].

CIO is a common constituent in numerous plants, exhibiting antimicrobial effects. It is widely used as flavor compound in foods, drinks, perfumes, and cosmetics. CIO and CI naturally occur in the spice cinnamon, which is derived from the bark of trees from the genus *Cinnamomum*. The main aromatic compound responsible for the flavor of cinnamon is CI, which is applied as a flavoring agent, an effective insecticide, and a potent fumigant endowed with antibacterial and antifungal properties [26]. Coumaric acid (COO, trans-3-(4-hydroxyphenyl)prop-2-enoic acid) is abundant in fruits (apples, pears, strawberries), vegetables (beans, potatoes, tomatoes), as well as in plant (tea) and animal (honey) products. COO prevents lipid peroxidation and endothelial cell death under oxidative stress, due to high levels of glucose and arachidonic acid, without affecting ROS production [27]. Coumaric aldehyde (CO, trans-3-(4-hydroxyphenyl)prop-2-enal) exhibits bacteriocidal effects, induces apoptosis in cancer cells, and inhibits cancer cell invasion and metastasis [28]. Ferulic acid (FEO, trans-3-(4-hydroxy-3-methoxyphenyl)prop-2-enoic acid) is found in a variety of plants such as vegetables, fruits, wheat, cereals, and barley. It possesses numerous beneficial bioactivities, including antioxidant, antiallergic, hepatoprotective, anticarcinogenic, antibacterial, and anti-inflammatory ones [29]. FEO is a potential neuroprotective agent as it ameliorates Alzheimer’s pathological symptoms by inhibiting β-amyloid aggregations in brain [30]. Sinapic acid (SIO, trans-3-(4-hydroxy-3,5-dimethoxyphenyl)prop-2-enoic acid) is a compound identified in fruits, vegetables (especially *Brassicaceae* family), cereal grains, oilseed crops, spices, and medicinal plants. SIO exhibits antianxiety, antioxidant, antimicrobial, anti-inflammatory, and anticancer activity [31]. It upregulates antioxidant levels, prevents the upregulation of cytokines (TNF-α, IL-6), and downregulates lipid peroxidation, resulting in a significant lowering of oxidative stress, and reducing apoptosis and inflammation [31].

**Figure 2:**
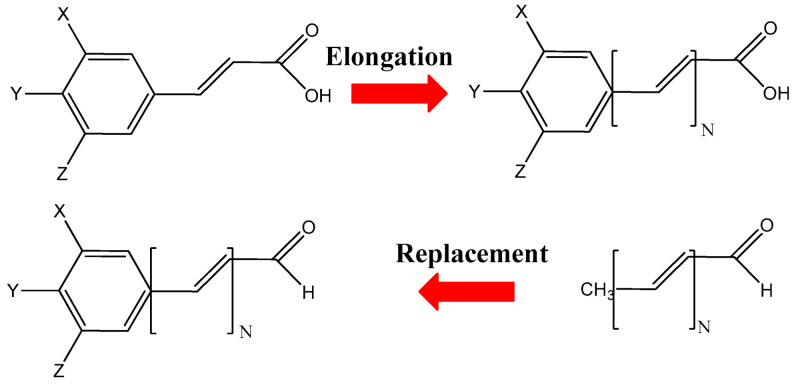
Enhancing bioactivity of the trans-phenylpropanoid acids through the elongation of the carbon chain with conjugated double bonds, and of the polyenals through the replacement of the inactive terminal methyl group by the substituted (X, Y, Z = H, OH, OCH_3_) benzene ring. Polyenals with N = 6–9 (tetradecahexenal, hexadecaheptenal, octadecaoctenal, eicosanonenal) have been identified in parrot feathers, whereas those with N = 6–14 have been identified in pearls, mollusk shells, and octocoral pigments.

A structural analysis of phenylpropanoid aldehydes and carboxylic acids reveals [32] a distinctive property of these compounds. Specifically, the benzene ring and the propenoic moiety function as two largely independent π-electron systems. Consequently, the direct electronic influence of the benzene ring on the vinyl group conjugated with the aldehyde or carboxyl group is restricted. This characteristic provides a foundation for the rational design of novel compounds formed through the combination of phenylpropanoids with polyene aldehydes and acids. These two classes may be integrated by extending the carbon chain via multiplication of the vinyl group, thereby generating an elongated, conjugated double-bond system. Conversely, the structure of polyene aldehydes can be modified by replacing the terminal, relatively unreactive methyl moiety with a benzene ring bearing functional substituents. As illustrated in Figure 2, these complementary strategies enable the incorporation of bioactive phenolic structures—widely distributed throughout the plant kingdom—into polyene aldehydes identified as constituents of parrot feather pigments. This approach thus provides a rational framework for structurally linking two classes of compounds, while preserving their intrinsic bioactivity and additionally imparting optical activity within the visible region. Consequently, the resulting compounds are anticipated to exhibit a broad spectrum of desirable properties, supporting their potential applications as multifunctional components in foods, pharmaceuticals, cosmetics, and dietary supplements.

The phenylpropanoids and their derivatives have been extensively studied through both experimental and theoretical approaches. Specifically, Kong et al. [33] investigated the adiabatic ionization energies (IE) of FEO and SIO, along with their corresponding anions, employing a combined DFT and semi-empirical AM1 method, labeled as B3LYP/6-31G(d)/AM1. Their findings indicate that the IE values of the anions exhibit strong correlation with experimental data regarding their reactions with NO_2_, while the IE values of the neutral species do not. This suggests a significant role of the phenolate anion in such reactions. Nsangou et al. [34] calculated the phenolic O–H bond dissociation enthalpy (BDE) of COO at the B3LYP/6-31+G(d,p) level of theory and compared the COO antioxidant activity with that of caffeic acid, a cinnamic acid derivative containing two hydroxyl groups in the benzene ring. Sebastian et al. [35] conducted quantum chemical calculations on the energies, geometries, and vibrational wavenumbers of FEO using the DFT method with the B3LYP and BLYP functionals, employing the 6-31G(d,p) basis set. The optimized geometrical parameters showed strong concordance with single crystal X-ray diffraction (XRD) data. Additionally, the calculated vibrational frequencies exhibited acceptable conformity with the experimentally measured infrared and Raman spectra of FEO. Further calculations for COO and FEO were performed [36] using the M05-2X functional [37] and the 6-311+G(d,p) basis set, in conjunction with the universal solvation model SMD [38], with pentyl ethanoate and water as solvents simulating lipid and aqueous environments, respectively. The study demonstrated [36] that environmental polarity significantly influences the efficacy of these compounds as radical scavengers. Specifically, the hydrogen transfer from the phenolic OH group was identified as the primary reaction mechanism in non-polar media, while the single electron transfer mechanism from the phenoxide anion was found to be responsible for the scavenging activity of COO and FEO in aqueous solutions. The thermodynamic activity parameters of COO, FEO, and SIO were determined, utilizing DFT at the B3LYP/6-311++G(2d,2p) level in both vacuum and solvent environments, including ethanol, water, and dimethyl sulfoxide media [39,40]. The research has demonstrated that COO, FEO, and SIO scavenge free radicals in the gas phase and non-polar media primarily through the HAT mechanism, whereas in polar media, they operate using the sequential proton loss electron transfer (SPLET) scenario. Additionally, it was proved that these compounds exhibit a significant capacity for complexation with heavy metal cations, which enhances their overall anti-radical potential [39,40]. Recently, Sherefedin et al. [41] calculated the dipole moments of FEO and SIO across various solvent polarities using the DFT and TD-DFT methods at the B3LYP/6-31G(d,p) level of theory. Their findings demonstrated that the dipole moments of FEO and SIO in the excited state are significantly higher than those in the ground state, indicating greater tendency of these molecules to undergo polarization under the influence of the polar solvents. However, the dipole moments calculated via computational methods showed discrepancies when compared to the experimental values, likely due to the influence of solvents during the measurements or the low accuracy of quantum chemical calculations. The spectroscopic properties of (methoxy, hexyloxy)phenyl polyenals, analogous to those depicted in Figure 2 with X=Z=H, Y=OCH_3_ or OC_6_H_13_, and containing N = 1, …6, 8 double bonds, have been investigated both experimentally and theoretically in [42]. This study demonstrated that increasing the solvent polarity induces a structural transition in the canonical polyene framework, shifting it from a more polymethine-like configuration to a state that is more polyene-like. The experimental observations, corroborated by theoretical calculations using the semi-empirical AM1-CI method and the SM5.4/A solvation model within the AMSOL software package, allowed for the determination of the HOMO (highest occupied molecular orbital) and LUMO (lowest unoccupied molecular orbital) energy gap across solvents of varying polarity.

A comprehensive examination of the aforementioned findings and the theoretical methods employed in their acquisition reveals several critical limitations: (i) the use of suboptimal basis sets, which may compromise the accuracy of the derived reactivity characteristics, (ii) the failure to determine the global chemical activity parameters beyond the ionization potential, electron affinity and HOMO–LUMO energies, and (iii) the dispersion of findings across various publications, thereby hindering a holistic view on the reactivity within the studied group of compounds. In light of the limitations mentioned, the primary objective of this study is to conduct more precise quantum chemical calculations surpassing those previously performed using the DFT method along poor basis sets, which may compromise the accuracy of calculated thermodynamic and reactivity descriptors of the selected natural phenylpropanoids. A secondary objective is to evaluate the activity descriptors of newly modeled synthetic analogs, which are derived from the combination of the selected phenylpropanoids with the polyene carboxylic acids and aldehydes. The specific structural features of the parent compounds suggest the potential to preserve their natural properties (e.g., antiradical, antimicrobial) while simultaneously imparting optical activity in the visible region, enabled by a polyene chromophore containing an appropriate number of conjugated double bonds. Consequently, the range of multifunctional components applicable in cosmetics, dietary supplements, pharmaceuticals, and food products may be substantially expanded.

## 2. Results and Discussion

To achieve the primary objectives of this work, quantum chemical calculations have been performed for the following compounds: (i) CIO, COO, FEO, and SIO acids, along with their corresponding aldehyde forms CI, CO, FE, and SI; and (ii) analogs of these compounds formed by their combination with tetradecahexenal (P6), hexadecaheptenal (P7), octadecaoctenal (P8), and eicosanonenal (P9) and their carboxylic forms PO6, PO7, PO8, and PO9. The thermodynamic and global chemical activity descriptors of all compounds considered have been determined using the DFT method at the B3LYP/QZVP level of theory, along with the C-PCM solvation model. The rationale for selecting these approaches is detailed in the Section 3, which also provides definitions of the thermodynamic descriptors and outlines the procedures used to determine the global chemical reactivity descriptors. Their physicochemical significance is further elaborated in the Supplementary Materials. In this study, the following descriptors are considered:

(i) Bond dissociation enthalpy BDE, adiabatic ionization potential AIP, proton dissociation enthalpy PDE, proton affinity PA, electron transfer enthalpy ETE, and the free Gibbs acidity G_acidity_ in the hydrophilic (water) solvent [43,44,45,46,47];
(ii) Ionization potential IP, electron affinity EA, energy gap ∆E, chemical potential μ, absolute electronegativity χ, molecular hardness η and softness S, electrophilicity index ω, the electro-donating ω^−^ and electro-accepting ω^+^ powers, and the Ra and Rd indexes [48,49,50,51,52,53,54,55].

**Figure 3:**
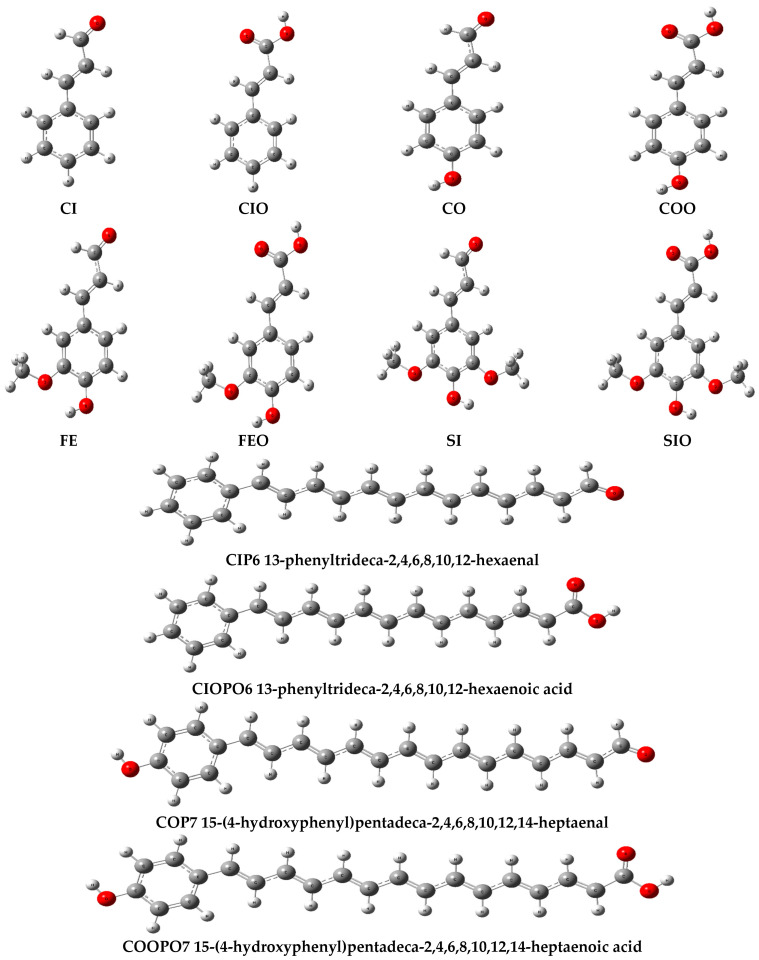

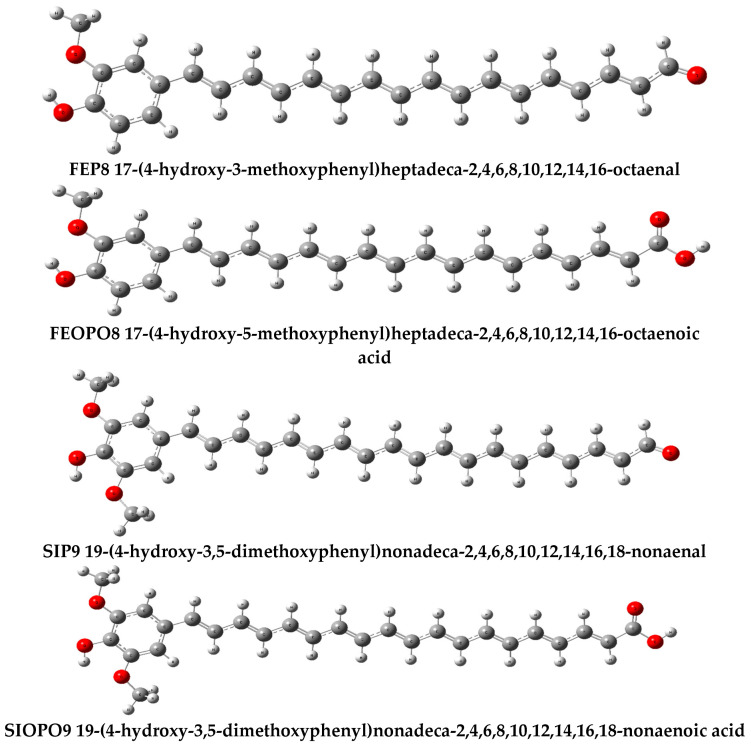
The optimized geometries of the trans-phenylpropanoids (aldehyde, acid): cinnamic (CI, CIO), coumaric (CO, COO), ferulic (FE, FEO), sinapic (SI, SIO) and their combinations with the polyenals: tetradecahexenal (CIP6), hexadecaheptenal (COP7), octadecaoctenal (FEP8), eicosanonenal (SIP9), and their carboxylic forms (CIOPO6, COOPO7, FEOPO8, SIOPO9). The optimization was performed at the DFT/B3LYP/QZVP level of the theory in water, using the C-PCM solvation model.

The radical-scavenging efficacies of the compounds investigated are determined by thermodynamic descriptors associated with the following deactivation mechanisms: BDE for hydrogen atom transfer (HAT), AIP and PDE for single electron transfer followed by proton transfer (SET-PT), PA and ETE for sequential proton loss electron transfer (SPLET), and G_acidity_ for transition metal chelation (TMC). These descriptors can be computed using the enthalpies of the cation H(R-H^●+^), radical H(R^●^), anion H(R^−^), the parent compound H(R-H), and the enthalpies of hydrogen H(H^●^), the electron H(e^−^), and the proton H(H^+^). A lower value of the calculated parameter indicates lower energy requirements for dehydrogenation (HAT), ionization (SET-PT), and deprotonation (SPLET) in the initial phase of radical deactivation. For two-stage processes, the sum of the relevant parameters (PA + ETE or AIP + PDE) should also be considered. The geometries of all compounds, including their cationic, anionic, and radical forms in the water medium, were optimized, following the procedure described in the Section 3. The optimized geometries of the neutral molecules considered are presented in Figure 3 and Figure S1.

The values of the thermodynamic descriptors reported in Table 1, Table 2, Table 3 and Table 4 reveal that in the hydrophilic phase, CO, FE, SI (PA1 > PA2), and COO (PA1 ≈ PA2, ETE1 > ETE2) scavenge radicals via the SPLET2 mechanism through deprotonation of the hydroxyl group attached to a benzene ring CO‒H, whereas CIO, FEO, and SIO (PA1 < PA2) do so through deprotonation of the carboxylic moiety O=CO‒H (SPLET1 mechanism). Only in the case of CI, the hydrogen detached from the aldehyde group O=C–H scavenges radicals via the HAT scenario. Since the PA parameters determining the activation of the SPLET mechanism and the proper active center (SPLET1 or SPLET2) satisfy the condition PA < BDE (with the exception of CI), the HAT mechanism is not energetically favored over SPLET for the molecules under consideration.

A comparison between the parameter values reported in Table 2, Table 3 and Table 4, calculated using the B3LYP/QZVP method, and those obtained by applying the B3LYP/6-311++G(2d,2p) level of theory [39,40] (values in brackets), reveals substantial discrepancies in the computed descriptors. For COO: BDE = 82.33 (85), PA = 44.08 (31), ETE = 84.34 (108), AIP = 115.71 (135), PDE = 12.72 (4), G_acidity_ = 287.42 (291) kcal mol^−1^. In the case of FEO: BDE = 78.91 (80.78), PA = 45.93 (30.35), ETE = 79.07 (80.31), AIP = 109.54 (106.84), PDE = 15.47 (7.17), G_acidity_ = 289.46 (289.91) kcal mol^−1^. For SIO: BDE = 74.97 (78), PA = 45.66 (30), ETE = 75.41 (103), AIP = 84.04 (104), PDE = 29.60 (2), G_acidity_ = 291.03 (290) kcal mol^−1^. These differences significantly influence the characteristics of the antiradical potency of COO, FEO, and SIO in the hydrophilic medium, although they do not alter the nature of the radical-scavenging mechanism. SPLET remains energetically preferred, with the hydrogen atom of the phenolic hydroxyl group being the most active in this process. On the other hand, it is noteworthy that the carboxyl and aldehyde groups of the compounds under investigation differentiate in their ability to chelate cations of transition metals (TMC mechanism), in contrast to the hydroxyl group, whose activity in this respect remains practically unchanged. The transition metal ions (especially Fe^2+^ and Cu^2+^), produce stable complexes that remove them from the reaction medium and contribute to the slowing down of the radical cascade reactions. Taking into account the activity of the hydroxyl group and the total energy of the SPLET2 process, represented by the PA2 + ETE2 parameter, it is possible to rank the compounds in terms of their radical-scavenging potentials (values in kcal mol^−1^ in parentheses): CO(128.88) ≈ COO(128.44) < FE(125.37) < SI(121.40). This ranking indicates that the antiradical activity of the tested compounds depends on the number of methoxy groups attached to the benzene ring. In the case of CIO, FEO, and SIO, and the deprotonation of the carboxylic moiety O=CO‒H via the SPLET1 scenario, characterized by the PA1 + ETE1 parameter, one gets: CIO(148.89) < FEO(142.20) < SIO(140.55). An analysis of the thermodynamic descriptors for the modeled combinations of phenylpropanoids with polyene aldehydes and acids suggests that these compounds exhibit greater antiradical activity than the parent compounds, which themselves are traditionally regarded as potent radical scavengers. Computational results indicate that in a hydrophilic (water) environment, radical-scavenging occurs via the SPLET mechanism, involving deprotonation of the carboxyl, aldehyde, and hydroxyl moieties, with polyene acids exhibiting stronger activity in this respect compared to polyene aldehydes. Taking into account the PA1,2 + ETE1,2 parameters (values in kcal mol^−1^ in parentheses), a radical-scavenging-activity ranking for the designed compounds can be established. For aldehyde derivatives: CIP6(121.69) < COP7(118.22) < FEP8(116.72) < SIP9(113.71), and for carboxylic derivatives: CIOPO6(131.66) < COOPO7(128.19) < FEOPO8(126.53) < SIOPO9(125.31). An examination of Table 2, Table 3 and Table 4 reveals that the elongation of the carbon chains CIO → CIOPO6, COO → COOPO7, FEO → FEOPO8, SIO → SIOPO9 has a marginal effect on the PA1 parameter values (they change about 1 kcal mol^−1^), while the ETE1 parameters exhibit significant changes (they decrease about 15–18 kcal mol^−1^). Since the difference in ETE1 values within the class (COO, FEO, SIO) is approximately 2 kcal mol^−1^, we conclude that the elongation of the carbon chain and the increase in the number of conjugated double bonds facilitate the activation of the second step of the SPLET1 mechanism, which involves the detachment of an electron from the carboxylic anionic form of the parent molecule. Consequently, the sum of PA1 + ETE1 decreases as the carbon chain increases, leading to an enhancement in antiradical activity of the carboxylic group through the SPLET1 scenario. A similar situation takes place during the elongation of the carbon chain in the aldehyde derivatives: CO → COP7, FE → FEP8, SI → SIP9, which also has a marginal effect on the PA2 parameters’ values (they change about 2 kcal mol^−1^), while the ETE2 parameters decrease about 10–13 kcal mol^−1^. Since the difference in ETE2 values within the class (CO, FE, SI) is 3 –4 kcal mol^−1^, we conclude that the elongation of the carbon chain facilitates the activation of the second step of the SPLET2 mechanism, which involves the detachment of an electron from the anionic aldehyde form of the parent molecule. Consequently, the sum of PA1 + ETE1 decreases as the carbon chain increases, leading to an enhancement in the antiradical activity of the hydroxylic group CO‒H through the SPLET2 scenario.

The global chemical reactivity descriptors presented in Tables S1–S4 in the Supplementary Materials demonstrate a clear dependence on the computational method employed (I, II, III), and is further described in Equations (5)–(7) in the Section 3. Specifically, the EA, S, χ, ω, ω^+^, ω^−^, Ra, and Rd parameters exhibit an increasing trend, while the IP, ∆E, and η parameters decrease as the method progresses from I to III. Nonetheless, regardless of the computational method used, the conclusions derived from the comparison of descriptors for different molecules within a given method remain consistent with the analysis of the thermodynamic descriptors. A marked increase in the reactivity of the modeled compounds, relative to the parent phenylpropene and polyene components, is observed. This is evidenced by a reduction in the values of the IP and ∆E parameters, alongside an increase in the EA descriptor. Additionally, the activity of the new compounds exhibits a stronger enhancement towards phenylpropanoids, than towards polyenoic acids and aldehydes. Given that the ranked polyenes (psittacofulvins) found in parrot feather pigments are potent radical scavengers, the modeled compounds display similar or slightly enhanced antiradical properties. Based on the values of the EA = −E(LUMO), IP = −E(HOMO), and ∆E (in brackets in [eV]) parameters generated by method I, a ranking of the chemical reactivity of the new compounds can be established: CIP6(3.0036, 5.4083, 2.4047) < COP7(3.0079, 5.1797, 2.1717) < FEP8(3.0469, 5.0738, 2.0270) < SIP9(3.0790, 4.9960, 1.9170) and CIOPO6(2.9021, 5.3802, 2.4781) < COOPO7(2.9113, 5.1571, 2.2458) < FEOPO8(2.9571, 5.0542, 2.0972) < SIOPO9(2.9946, 4.9786, 1.9840). This ranking indicates that the chemical reactivity of the modeled compounds increases under structural elongation. The collection of the above specified parameters and the frontier orbitals associated with the HOMO–LUMO energies are presented in Figure S2. A small energy gap characterizes a soft molecule that is less stable and more reactive. A small IP value indicates a greater tendency of a compound to participate in the chemical reaction related to electron transfer, whereas large values of EA characterize a greater capacity of a molecule to accept electrons and to convert it to the anionic form. Although the increase in EA and the decrease in IP values reported above are negligible, the change in the energy gap ∆E is substantial and indicates an increase in the activity of the analyzed compounds according to the ranking. Comparison of analogous parameters for the aldehyde and acid forms leads to the interesting conclusion that aldehydes show higher chemical activity than their carboxylic analogs. The parameters determined using methods II and III yield identical conclusions. The descriptors (method I) characterizing the electro-donating ω^−^ and electro-accepting ω^+^ power of a radical scavenger steadily increase from ω^−^ = 9.6097, 9.1457; ω^+^ = 5.4038, 5.0045 [eV] (CIP6, CIOPO6), up to ω^−^ = 10.6419, 10.1280; ω^+^ = 6.6044, 6.1414 [eV] (SIP9, SIOPO9). This indicates that the antioxidant activity of the modeled compounds diminishes as their reactivity increases, in contradistinction to their anti-reductant activity, which increases. This effect is closely linked to the elongation of the polyene chain attached to the benzene ring. The conclusions presented above are consistent with the acceptance Ra and donation Rd indexes, which increase from Ra = 1.5889, 1.4711; Rd = 2.7695, 2.6358 (CIP6, CIOPO6), up to Ra = 1.9414, 1.8052; Rd = 3.0670, 2.9189 (SIP9, SIOPO9). Consequently, all analogs considered are more effective electron acceptors than F (Ra = 1) and more effective electron donors than Na (Rd = 1). We conclude that their reactivity is greater than that attributed to astaxanthin [55]: Ra = 0.94, Rd = 2.10, ω^+^ = 3.21, ω^−^ = 7.27, which is recognized as the most effective electron acceptor among the pigments identified in nature [55]. Analysis of the remaining parameters of chemical activity (χ, η, S) reveals a weak dependence of χ and η on the elongation of the carbon chain, in contradistinction to S which increases significantly. This proves that structural elongation affects the hardness S of the molecule, i.e., the susceptibility to deformation or polarization of the electron cloud under the influence of external factors (reagents).

An issue of particular importance for the practical applications of the modeled compounds, is whether the incorporation of a polyene chromophore affects the length and bond order of the C–C bond separating the benzene ring from the polyene chain. The computational results presented in Table 5 clearly indicate that such an effect is absent or only marginal.

Thus, the modeled synthetic analogs exhibit a separation between the benzene ring and the polyene chromophore that is identical or comparable to that observed in the parent phenylpropanoids. This suggests a high probability that the valuable properties of natural phenylpropanoids (e.g., antiradical, antimicrobial) may be transferred to their synthetic analogs. Verification of this hypothesis, however, requires experimental laboratory studies.

The modeled compounds contain a polyene moiety that enables the absorption of radiation within the visible region. This hypothesis is supported by time-dependent density functional theory (TD-DFT) calculations performed for the acidic forms CIOPO6, COOPO7, FEOPO8, and SIOPO9, as well as for the corresponding aldehyde forms CIP6, COP7, FEP8, and SIP9. On the basis of fully optimized ground-state structures in an aqueous solution, TD-DFT calculations were carried out at the B3LYP/QZVP level of theory using the C-PCM solvation model in order to determine the low-lying electronic excited states. The obtained results, including oscillator strengths (f) and maximum absorption wavelengths (λ_max,_ in [nm]), predict the dominant electronic transitions at λ_max_ = 521.21 nm (f = 2.9439) for CIOPO6 and 534.25 [nm] (f = 2.9444) for CIP6; 579.93 [nm] (f = 3.2080) for COOPO7 and 596.67 nm (f = 3.1873) for COP7; 625.94 nm (f = 3.4632) for FEOPO8 and 644.90 nm (f = 3.3142) for FEP8; and 666.45 nm (f = 3.7342) for SIOPO9 and 687.22 nm (f = 3.6587) for SIP9. These results indicate that the investigated compounds may act not only as efficient radical scavengers but also as potential colorants, thereby broadening the scope of their possible practical applications.

## 3. Materials and Methods

The newly designed chemical compounds are a combination of the selected phenylpropanoids and polyene aldehydes (or acids). For the latter component, the quantum chemical calculations of the reactivity descriptors were already performed [56] using the DFT/B3LYP method, QZVP basis set, and the C-PCM solvation model. This approach utilizes Becke’s [57] exchange functional in conjunction with the Lee–Yang–Parr [58] functional (B3LYP), the Weigend–Ahlrichs valence quadruple-zeta polarization basis set (QZVP) [59], and the conductor-like polarizable continuum model (C-PCM) [60]. The selection of an appropriate basis set and functional may be guided by their ability to reproduce available experimental data; in the absence of such data, the primary criterion for assessing the adequacy of the model is the minimization of the electronic energy. To determine the optimum calculation method for the phenylpropene component, the test calculations for the simplest CI molecule in the water environment were conducted, employing DFT/ B3LYP method, the C-PCM model, and the correlation-consistent polarized-valence quadruple zeta basis sets (cc-pVQZ and aug-cc-pVQZ) introduced by Dunning [61]. These calculations yielded the energy values of E(cc-pVQZ) = −423.170845 [Ha], E(aug-cc-pVQZ) = −423.173149 [Ha], and E(QZVP) = −423.178463 [Ha], with QZVP providing the lowest energy at the B3LYP theory level. Energy differences E(cc-pVQZ) − E(aug-cc-pVQZ) = 1.45 kcal mol^−1^ indicate that the inclusion of diffuse functions slightly lowers the electronic energy of the molecule, in contrast to the QZVP basis set, which does not incorporate diffuse functions, yet leads to a significant reduction in energy as E(cc-pVQZ) − E(QZVP) = 4.78 kcal mol^−1^. Notably, the energy difference of 1.45 kcal mol^−1^ only marginally exceeds the generally accepted threshold of 1 kcal mol^−1^ for thermochemical accuracy in computational calculations. Since diffusive effects are expected to play a significant role in the case of ions, calculations were performed for the Cl^−^ anion yielding: E(cc-pVQZ) − E(aug-cc-pVQZ) = 2.10 kcal mol^−1^, E(cc-pVQZ) − E(QZVP) = 5.32 kcal mol^−1^. These calculations demonstrated that the inclusion of the QZVP basis set significantly lowers the electronic energy in comparison with the aug-cc-pVQZ basis set. Calculations carried out for CIP6 further revealed that a similar energetic gain occurs upon elongation of the polyene chain. In this case, the differences in energy amount to E(cc-pVQZ) − E(aug-cc-pVQZ) = 2.47 kcal mol^−1^ and E(cc-pVQZ) − E(QZVP) = 8.82 kcal mol^−1^, respectively. Subsequent test calculations demonstrated that altering the functional from B3LYP to M06-2X [62], and subsequently to BHandHLYP [63], resulted in an increase in the calculated energy of CI, E(M06-2X) = −422.994455 [Ha] and E(BHandHLYP) = −422.912987 [Ha] in comparison to E(B3LYP) = −423.178463 [Ha], whereas changing the solvation model from C-PCM to the popular IEF-PCM (Integral Equation Formalism-Polarizable Continuum Model) [64] had only a minor effect on the calculated energy, at E(IEF-PCM) = −423.178409 [Ha]. Consequently, the method B3LYP/QZVP and model C-PCM were used in calculations for all the phenylpropanoids and their combinations with the polyene aldehydes (acids). To calculate the enthalpies H(R-H^●+^), H(R^●^), H(R^−^), and H(R-H), the Gibbs free energies G(R^−^) and G(R-H) which are indispensable in the determination of thermodynamic descriptors, and the energies E^0^(R-H^●+^), E^0^(R-H^●−^), E(R-H^●+^), E(R-H^●−^), and E(R-H) applied in the calculation of the chemical activity descriptors, we employed the DFT method implemented in the Gaussian vs. 16 software (Gaussian Inc., Wallingford, CT, USA) [65]. The input structures were constructed by taking advantage of the Gauss View-6.1 graphical interface (Gaussian Inc., Wallingford, CT, USA) [65], whereas the calculations were carried out in the Supercomputing and Networking Center of Poznań. Since the computations for the compounds studied at the B3LYP/QZVP level are time-consuming and their convergence depends on the initial geometry input, in the first stage, the calculations were carried out for various phenylpropanoid conformers with the goal of identifying those with the lowest energy.

The optimized geometries of the compounds corresponding to the lowest energy values are presented in Figure 3 and Figure S1. Non-planar conformers, characterized by the methoxy group positioned above and below the plane of the benzene ring, were excluded due to their relatively higher energy values compared to the planar structures. The frontier orbitals and corresponding HOMO–LUMO energies of the modeled compounds are illustrated in Figure S2, whereas Figure S3 depicts the spin density distributions for their radical forms. After identifying the lowest-energy conformers, calculations were conducted on their modified forms, obtained by elongating the conjugated double-bond system. The structures of both the original phenylpropanoids and their modified derivatives were optimized within the three stages: (i) the determination of an approximate geometry at the B3LYP/6311++G(d,p) level of the theory; (ii) the geometry refinement at the B3LYP/cc-pVQZ level; and (iii) the final calculation at the B3LYP/QZVP level in the water medium. Thus, optimization was performed at each stage, with the geometry determined at the lower level serving as the starting point for optimization at the higher level. The enthalpies H and free Gibbs energies G obtained within this scheme were subsequently employed in the calculation of the thermodynamic descriptors, defined as follows:

(1) $$\mathrm{BDE}\;=\;H(R^{•})\;+\;H(H^{•})\;-\;H(R\text{-}H)\;(HAT)$$

(2) $$\mathrm{PA}=H(R^{-})+H(H^{+})\;-\;H(R\text{-}H)\;\;\;\;\;\mathrm{ETE}=H(R^{•})+H(e^{-})\;-\;H(R^{-})\;(\mathrm{SPLET})$$

(3) $$\mathrm{AIP}=H(R\text{-}H^{•+})+H(e^{-})\;-\;H(R\text{-}H)\;\;\;\;\;\mathrm{PDE}=H(R^{•})+H(H^{+})\;-\;H(R\text{-}H^{•+})\;(\mathrm{SET}\text{-}\mathrm{PT})$$

(4) $$G_{\mathrm{acidity}}=G\left(R^{-}\right)\;-\;G\left(R\text{-}H\right)(TMC)$$

Here, H(R^●^), H(R^−^), G(R^−^), H(R-H^●+^), H(R-H), and G(R-H) denote enthalpies and Gibbs energies of the radical, anion, cation, and the neutral parent molecule, whereas H(H^●^), H(H^+^), and H(e^−^) represent the enthalpies of the hydrogen, proton, and electron. In calculations, we used the values (in Hartree [Ha] unit) of the enthalpies in water [43]: H(e^−^)_aq_ = −0.03879545, H(H^+^)_aq_ = −0.38690958, H(H^●^)_aq_ = −0.49916356. They can be calculated using the following relationships:

H(e^−^)_sol_ = H(e^−^) + ∆_sol_H(e^−^), H(H^+^)_sol_ = H(H^+^) + ∆_sol_H(H^+^), H(H^●^)_sol_ = H(H^●^) + ∆_sol_H(H^●^)

in which ([kJ mol^−1^] unit) ∆_aq_H(e^−^) = −105, ∆_aq_H(H^+^) = −1022, and ∆_aq_H(H^●^) = −4.0, which are the solvation corrections recommended by Rimarčik et al. [43].

The global descriptors of chemical reactivity, as defined in the Supplementary Materials, depend on the ionization potential (IP) and electron affinity (EA). These parameters can be determined using three different methods. Based on the energy of the HOMO and LUMO orbitals, as well as the Koopmans’ theorem [48] for the closed shell molecules, one may define (method I):

(5) $${\mathrm{IP}}^{I}{=-E}_{\mathrm{HOMO}}\;\;\;\;\;{\mathrm{EA}}^{I}{=-E}_{\mathrm{LUMO}}$$

As Koopmans’ theorem is a crude approximation, the IP and EAM correspond rather to the difference between the total energies of the M‒1 and M-electron states [49]. Therefore, IP and EA can be calculated using the formula (method II) [49]:

(6) $${\mathrm{IP}}^{\mathrm{II}}{=E}_{M-1}(G_{M})-E_{M}(G_{M})\;\;\;\;\;{\mathrm{EA}}^{\mathrm{II}}{=E}_{M}(G_{M})-E_{M+1}(G_{M})$$

in which E_M−1_(G_M_) = E^0^(R-H^●+^) is the vertical energy of the M−1 electron systems (cation), calculated at the fixed (single-point) geometry G_M_ of the neutral molecule endowed with the energy E_M_(G_M_) = E(R-H). Method III of determining IP and EA is based on the equation [49]

(7) $${\mathrm{IP}}^{\mathrm{III}}{=E}_{M-1}(G_{M-1})-E_{M}(G_{M})\;\;\;\;\;{\mathrm{EA}}^{\mathrm{III}}{=E}_{M}(G_{M})-E_{M+1}(G_{M+1})$$

in which E_M−1_(G_M−1_) = E(R-H^●+^) denotes the optimized energy of the M−1 electron system calculated for the geometry G_M−1_ of the cation. Method III usually produces IP and EA values closer to those derived from experiments, in comparison to methods I and II, which do not take into account geometry relaxation after the electron transfer.

**Figure 4:**
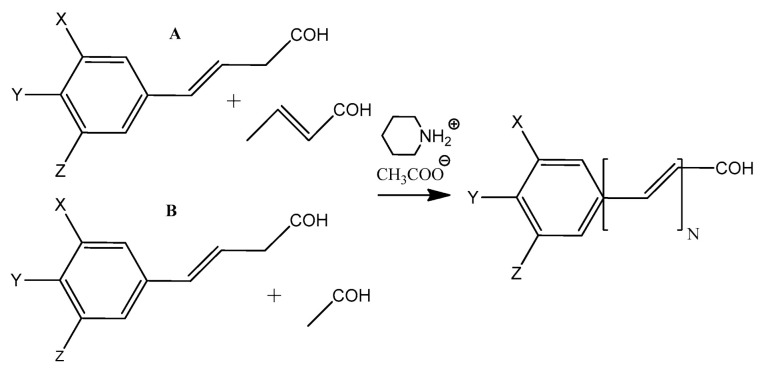
The proposed synthetic route for compounds CIP6, COP7, FEP8, and SIP9, featuring an odd (path (**A**)) and even (path (**B**)) number of double bonds.

An important aspect to consider is the method of synthesizing the modeled compounds. In the case of the aldehyde forms, one may adopt synthetic strategies analogous to those employed in the preparation of polyenal aldehydes (e.g., psittacofulvins) [66]. Aldehydes containing an odd number of conjugated double bonds can be synthesized via the condensation of phenylpropanoid aldehydes with crotonaldehyde in the presence of the catalyst piperidinium acetate (reaction A in Figure 4). In contrast, aldehydes with an even number of double bonds may be obtained through the condensation of phenylpropanoid aldehydes with acetaldehyde, also in the presence of piperidinium acetate (reaction B in Figure 4).

## 4. Conclusions

Calculations of thermodynamic and chemical descriptors offer a fundamental advantage over the determination of kinetic characteristics, as they are independent of the type of scavenged radical. They enable the preliminary screening of modeled compounds in terms of their desired (enhanced) activity and potential applicability. Only at a subsequent stage of research can their targeted activity toward the neutralization of specific radicals be determined. It is made possible through kinetic calculations of activation barriers, transition states, and reaction efficiencies. Such calculations should be preceded by the synthesis of the modeled compounds and the experimental verification of their activity, thereby allowing for a direct comparison with the results of kinetic computations.

The thermodynamic descriptors for the modeled combinations of the selected phenylpropanoids with polyene aldehydes (acids) indicate that these compounds demonstrate enhanced antiradical activity compared to their parent components, which are already traditionally regarded as potent radical scavengers. The computational method employed yields reliable values of the BDE descriptor for COO, FEO, and SIO, in agreement with the values reported in the literature [67]. It also reproduces the ranking of radical-scavenging activity (COO < FEO < SIO), indicating that sinapic acid is a highly effective radical scavenger compared to coumaric and ferulic acids, which exhibit lower activity in this respect. Computational analysis reveals that the synthetic phenylpropanoids considered primarily scavenge radicals via the SPLET scenario in aqueous (hydrophilic) environments. This mechanism involves the deprotonation of carboxyl, aldehyde, or hydroxyl functional groups, with the hydroxyl group exhibiting the highest activity in this regard. The elongation of the carbon chain, incorporating a system of conjugated double bonds, has a minor effect on the PA parameter values, while significantly altering the ETE parameters. Thus, the elongation of the carbon chain and the increase in the number of conjugated double bonds enhance the activation of the second step in the SPLET mechanism, leading to an improvement in the antiradical activity of the designed scavengers. The conclusion outlined above aligns with the spin density distribution (see Figure S3) observed in the radical forms of the modeled compounds, indicating charge delocalization across the benzene ring and carbon chain. This charge delocalization enhances the chemical stability of the radical species generated in the SPLET scenario, consequently influencing the efficacy and potency of the designed compounds as radical scavengers. Furthermore, this delocalization facilitates electron detachment from the parent (anionic) molecule, as evidenced by the low ETE parameter values characterizing the second stage of the SPLET mechanism. The computed global chemical reactivity parameters corroborate the findings derived from the thermodynamic descriptors. They demonstrate a substantial increase in the chemical reactivity of the modeled compounds relative to their parent phenylpropene and polyene components. It is evidenced by a decrease in the IP and energy gap ∆E values, alongside an increase in the EA parameter. Consequently, the activity of the new, designed compounds exhibits a stronger enhancement towards phenylpropanoids and polyenoic acids (aldehydes) recognized as potent radical deactivators. The proposed approach for the modeling of novel phenylpropanoid derivatives is generalizable and allows for the incorporation of an arbitrary number of conjugated double bonds into the carbon chain. Consequently, new synthetic analogs with N = 2, 3, 4, 5, or more double bonds, as well as other functional groups X, Y, and Z (Figure 2) attached to the benzene ring, can be introduced. This approach significantly expands the range of bioactive compounds with strong antiradical properties [25,26,27,28,29,30,31,32,68,69,70,71] and thus has considerable applicative potential. Modern components of food and cosmetic formulations are expected to be multifunctional to reduce toxicity and minimize the risk of interactions between individual constituents. The modeled compounds, in addition to their radical-scavenging activity (associated with phenylpropanoids) and antimicrobial properties (associated with psittacofulvins) inherited from their structural motifs, are also capable of absorbing light in the visible range. Consequently, they may serve as active colorants, typically added to food products and colored cosmetic formulations.

## Figures and Tables

**Table 1:** The thermodynamic descriptors (kcal mol^−1^) of cinnamaldehyde (CI), cinnamic acid (CIO), tetradecahexenal (P6), tetradecahexenoic acid (PO6), and their combinations (CIP6, CIOPO6) presented in Figure 2 and Figure 3. The calculations were performed in the water medium at the B3LYP/QZVP theory level, using the C-PCM solvation model. Active hydrogens from the moieties 1. O=C‒H (aldehyde), and 2. O=CO‒H (acid) have been taken into account. The values of P6 and PO6 have been reported in [56].

Descriptor	CI	CIP6	P6	CIO	CIOPO6	PO6
BDE	84.67	75.59	99.17	102.79	85.56	86.15
PA	90.20	70.41	67.64	42.85	43.88	44.10
ETE	40.56	51.28	77.63	106.04	87.78	88.15
PA+ETE	130.77	121.69	145.27	148.89	131.66	132.25
AIP	127.47	93.91	89.55	126.42	93.28	94.58
PDE	3.29	27.78	55.71	22.47	38.38	37.66
G_acidity_	332.61	268.08	311.24	285.62	286.20	286.42
Mechanism	HAT1	SPLET1	SPLET1	SPLET1	SPLET1	SPLET1

**Table 2:** The thermodynamic descriptors (kcal mol^−1^) of coumaric aldehyde (CO), coumaric acid (COO), hexadecaheptenal (P7), hexadecahepteoic acid (PO7), and their combinations (COP7, COOPO7) presented in Figure 2 and Figure 3. The calculations were performed in the water medium at the B3LYP/QZVP theory level, using the C-PCM solvation model. Two active hydrogens from the moieties 1. O=C‒H, O=CO‒H, and 2. CO‒H (phenolic) have been taken into account. The values of P7 and PO7 parameters have been reported in [56].

Descriptor	CO	COP7	P7	COO	COOPO7	PO7
BDE1 BDE2	86.48 82.78	75.19 72.12	75.69 -	98.58 82.33	82.09 71.97	84.23 -
PA1 PA2	96.12 43.04	70.53 45.13	72.51 -	43.85 44.08	44.08 45.58	44.11 -
ETE1 ETE2	36.46 85.84	50.76 73.09	49.27 -	100.82 84.34	84.10 72.48	86.22 -
PA1+ETE1 PA2+ETE2	132.57 128.88	121.28 118.22	121.78 -	144.67 128.42	128.19 118.06	130.33 -
AIP	116.75	88.54	92.04	115.71	88.05	91.43
PDE1 PDE2	15.82 12.13	32.74 29.68	29.74 -	28.97 12.72	40.13 30.01	38.90 -
G1_acidity_ G2_acidity_	337.82 286.34	313.21 288.54	315.59 -	286.49 287.42	313.21 288.54	286.49 -
Mechanism	SPLET2	SPLET2	SPLET1	SPLET2	SPLET1	SPLET1

**Table 3:** The thermodynamic descriptors (kcal mol^−1^) of ferulic aldehyde (FE), ferulic acid (FEO), octadecaoctenal (P8), octadecaoctenaenoic acid (PO8), and their combinations (FEP8, FEOPO8) presented in Figure 2 and Figure 3. The calculations were performed in the water medium at the B3LYP/QZVP theory level, using the C-PCM solvation model. Two active hydrogens from the moieties 1. O=C‒H, O=CO‒H, and 2. CO‒H (phenolic) have been taken into account. The values of P8 and PO8 parameters have been reported in [56].

Descriptor	FE	FEP8	P8	FEO	FEOPO8	PO8
BDE1 BDE2	86.26 79.27	74.60 70.63	74.99 -	96.11 78.91	80.44 70.53	82.59 -
PA1 PA2	96.44 44.76	68.90 46.76	70.47 -	43.88 45.93	44.16 47.22	44.12 -
ETE1 ETE2	35.91 80.60	51.80 69.97	50.60 -	98.32 79.07	82.37 69.40	84.57 -
PA1+ETE1 PA2+ETE2	132.35 125.37	120.70 116.72	121.07 -	142.20 125.00	126.53 116.62	128.69 -
AIP	110.49	86.16	89.50	109.54	85.70	88.94
PDE1 PDE2	21.86 14.87	34.54 30.57	31.59 -	32.67 15.47	40.83 30.92	39.75 -
G1_acidity_ G2_acidity_	337.99 288.27	311.21 290.95	313.72 -	286.04 289.46	286.81 291.26	286.50 -
Mechanism	SPLET2	SPLET2	SPLET1	SPLET1	SPLET1	SPLET1

**Table 4:** The thermodynamic descriptors (kcal mol^−1^) of sinapic aldehyde (SI), sinapic acid (SIO), eicosanonenal (P9), eicosanonenaoic acid (PO9), and their combinations SIP9 and SIOPO9, presented in Figure 2 and Figure 3. The calculations were performed in the water medium at the B3LYP/QZVP theory level, using the C-PCM solvation model. Two active hydrogens from the moieties 1. O=C‒H, O=CO‒H, and 2. CO‒H (phenolic) have been taken into account. The values of P9 and PO9 parameters have been reported in [56].

Descriptor	SI	SIP9	P9	SIO	SIOPO9	PO9
BDE1 BDE2	86.23 75.30	74.05 67.61	74.41 -	94.45 74.97	79.22 67.54	81.16 -
PA1 PA2	96.38 44.42	67.55 46.24	68.85 -	43.92 45.66	44.15 46.78	44.13 -
ETE1 ETE2	35.95 76.98	52.59 67.46	51.66 -	96.63 75.41	81.16 66.86	83.13 -
PA1+ETE1 PA2+ETE2	132.33 121.40	120.14 113.71	120.51 -	140.55 121.07	125.31 113.64	127.26 -
AIP	107.52	86.67	87.43	106.52	84.04	86.93
PDE1 PDE2	24.81 13.88	33.47 27.03	33.08 -	34.03 14.55	41.27 29.60	40.33 -
G1_acidity_ G2_acidity_	337.62 287.65	310.47 290.43	312.26 -	286.78 289.29	287.16 291.03	286.47 -
Mechanism	SPLET2	SPLET2	SPLET1	SPLET1	SPLET1	SPLET1

**Table 5:** The C–C bond length (in Å) linking the benzene ring to the polyene component in natural and synthetic phenylpropanoids. The calculations were performed in the water medium at the B3LYP/QZVP theory level, using the C-PCM solvation model.

Compound	C–C	Compound	C–C
CI	1.456	FE	1.449
CIP6	1.456	FEP8	1.453
CIO	1.458	FEO	1.452
CIOPO6	1.457	FEOPO8	1.454
CO	1.448	SI	1.448
COP7	1.452	SIP9	1.453
COO	1.453	SIO	1.452
COOPO7	1.453	SIOPO9	1.454

## Data Availability

The original contributions presented in this study are included in the article/Supplementary Materials. Further inquiries can be directed to the corresponding author.

## Supplementary Materials

The following supporting information can be downloaded at: https://www.mdpi.com/article/10.3390/ijms27083378/s1.

## Abbreviations

The following abbreviations are used in this manuscript:

ACE	Angiotensin Converting Enzyme
AIP	Adiabatic Ionization Potential
BDE	Bond Dissociation Enthalpy
CI	Cinnamaldehyde
CIO	Cinnamic Acid
CO	Coumaric Aldehyde
COO	Coumaric Acid
CPET	Concerted Proton-Electron Transfer
DFT	Density Functional Theory
EA	Electron Affinity
ETE	Electron Transfer Enthalpy
FE	Ferulic Aldehyde (FE)
FEO	Ferulic Acid
HAT	Hydrogen Atom Transfer
HOMO	Highest Occupied Molecular Orbital
IP	Ionization Potential
LUMO	Lowest Occupied Molecular Orbital
P6	Tetradecahexenal
P7	Hexadecaheptenal
P8	Octadecaoctenal
P9	Eicosanonenal
PA	Proton Affinity
PCET	Proton-Coupled Electron Transfer
PDE	Proton Dissociation Enthalpy
PO6	Tetradecahexenoic Acid
PO7	Hexadecahepteoic Acid
PO8	Octadecaoctenaenoic Acid
PO9	Eicosanonenaoic Acid
RAF	Radical Adduct Formation
RNS	Reactive Nitrogen Species
ROS	Reactive Oxygen Species
SET	Single Electron Transfer
SET-PT	Single Electron Transfer Followed by Proton Transfer
SI	Sinapic Aldehyde
SIO	Sinapic Acid
SPLET	Sequential Proton Loss Electron Transfer
TD-DFT	Time Dependent Density Functional Theory
VIP	Vertical Ionization Potential
