# Supplementary material for: Rational Design of Conjugated Phenylpropanoid–Polyene Hybrids: Density Functional Theory Insights into Antiradical and Optical Properties
Source: Int J Mol Sci. 2026 Apr 9;27(8):3378. doi: 10.3390/ijms27083378 (PMC13115693; doi:10.3390/ijms27083378)
Supplement: Supplementary file 1 [file ijms-27-03378-s001.zip › ijms-4212943-supplementary.pdf]

Supplementary Materials

Rational Design of Conjugated Phenylpropanoid–Polyene Hybrids: Density Functional Theory Insights into Antiradical and Optical Properties

Marcin Molski

Quantum Chemistry Department
Faculty of Chemistry
Adam Mickiewicz University
ul. Uniwersytetu Poznańskiego 8, 61-614 Poznań, Poland
mamolski@amu.edu.pl

1. Scavenging Mechanisms

Free radicals can be deactivated by the R─H compound via the three fundamental mechanisms specified below.

HAT (*Hydrogen Atom Transfer*)

$$R{-}H + X^{\bullet} \rightarrow R^{\bullet} + X{-}H$$

This mechanism is widely applied in processes in which the hydrogen atom is transferred from the basic compound to the reactive intermediate. The antiradical potency of a compound is related to the low bond dissociation enthalpy (BDE) of the N–H bond in amines, S–H in organosulfur compounds, O–H in polyphenols, alcohols, carboxylic acids, and O=C–H in aldehydes. Hence, crucial for calculating the BDE parameter is the reaction

$$R{-}H \rightarrow R^{\bullet} + H^{\bullet}$$

initiating the HAT process, which allows defining [43] the BDE parameter (1) presented in the Materials and Methods section.

SPLET (*Sequential Proton Loss Electron Transfer*)

$$R{-}H \rightarrow R^{-} + H^{+}$$

$$R^{-} + X^{\bullet} + H^{+} \rightarrow R^{\bullet} + X{-}H$$

The second step of the reaction above specified is initiated by the reaction

$$R^{-} \rightarrow R^{\bullet} + e^{-}$$

The SPLET process is therefore characterized [43] by the proton affinity (PA) and electron transfer enthalpy (ETE) calculated according to the formulae (2). If the reactions specified above are not sequential and involve the concerted shift of a single electron and a single proton, they are called Concerted Proton-Electron Transfer (CPET) [44]. This pathway belongs to the wide class of a Proton-Coupled Electron Transfer (PCET) [45], which involves the shift of electrons and protons from one molecule (atom) to another

SET-PT (*Single Electron Transfer Followed by Proton Transfer*)

$$R{-}H + X^{\bullet} \rightarrow R{-}H^{\bullet+} + X^{-}$$

$$R{-}H^{\bullet+} \rightarrow R^{\bullet} + H^{+}$$

This scenario involves the transfer of an electron from the parent compound

$$R{-}H \rightarrow R{-}H^{\bullet+} + e^{-}$$

generating a cation radical $R{-}H^{\bullet+}$ and, in the next step, the proton transfer from the cation radical producing parent agent in the radical form $R^{\bullet}$. These two stages are characterized by the adiabatic ionization potential (AIP) and the proton dissociation enthalpy (PDE) [43] calculated from the formulae (3). AIP is determined using the enthalpies of the optimized cationic state and the optimized neutral molecule. The Vertical Ionization Potential (VIP) defined as the difference between the energy of the optimized neutral structure and that of the corresponding cationic system at the neutral geometry is not considered in this work.

TMC (*Transition Metals Chelation*)

This mechanism is related to the chelation of the transition metal ions (especially Fe^{2+} and Cu^{2+}), which produces stable complexes that remove them from the reaction medium and contribute to the slowing down of the radical cascade reactions. Vital for this mechanism is the dissociation decay

$$R{-}H \rightarrow R^{-} + H^{+}$$

The parameter characterizing TMC in solvents is the free Gibbs acidity [43], which allows defining the $G_{acidity}$ parameter (4) presented in the Materials and Methods section. A lower value of this parameter indicates a greater ability to chelate metals and, consequently, a greater potency to slow down radical processes.

RAF (*Radical Adduct Formation*)

In this scenario, radicals are deactivated in the reaction [47]

$$R{-}H + X^{\bullet} \rightarrow R{-}XH^{\bullet}$$

which is characterized by the enthalpy or Gibbs free energies formation calculated for a given radical and an antioxidant considered. Hence, this pathway is characterized by the descriptor depending on the radical type. Consequently, it will not be considered here.

In calculations of the BDE, PDE, AIP, ETE, PA parameters, we used the values of the electron, proton and hydrogen enthalpies in water recommended by Rimarčik et al. [43].

2. Chemical Activity Global Descriptors

Ionization potential IP and electron affinity EA

IP describes the minimum energy required to remove an electron from the molecule's ground state and move it to infinity. A small value of IP indicates a greater tendency of the molecule to participate in the chemical reaction related to electron transfer. The best radical scavengers are endowed with low IP values. EA expresses the ability of a molecule to accept an electron and produce an anion. Due to the fact that radicals scavenging might act either via donating or accepting electrons, the EA descriptor is useful to characterize the capacity of a compound to accept electrons. On the basis of the energies of the HOMO and LUMO orbitals, together with

equations (5), (6), and (7) presented in the Materials and Methods section, methods I, II, and III can be employed to determine IP and EA. These values are subsequently used to calculate the remaining chemical reactivity descriptors according to equations (S1)–(S8) provided below.

Energy gap

The energy gap (usually expressed in electron volts [eV]) can be calculated by taking advantage of the relationship

$$\Delta E = IP - EA \tag{S1}$$

A large energy difference defines a *hard* molecule that is more stable and less active, while a small energy gap defines a *soft* molecule that is less stable and more reactive. The energy gap can be calculated using methods I, II, and III, so based on Koopmans' theorem [48], ΔE is approximately reproduceable by the difference between the LUMO and HOMO energies [49].

Chemical hardness

$$\eta \approx \frac{IP - EA}{2} \tag{S2}$$

It describes [50,51] the susceptibility of a molecule to deformation or polarization of its electron cloud under the influence of external factors, such as reagents or applied fields.

Chemical softness

$$S \approx \frac{1}{IP - EA} \tag{S3}$$

The inverse of chemical hardness characterizes molecules with the high susceptibility to deformation and polarization of the electron cloud [50,51].

Chemical potential

$$\mu \approx -\frac{(IP + EA)}{2} \tag{S4}$$

Employed to describe the thermodynamic activity of molecules and used, for example, in the derivation of the phase equilibrium constants [50].

Electronegativity

$$\chi = -\mu \approx \frac{IP + EA}{2} \tag{S5}$$

It characterizes a tendency to attract electrons that create the chemical bond [52]. The common electron pair is shifted towards the atom with a high value of χ, which is accompanied by the formation of a polar or ionic bond.

Electrophilicity Index

$$\omega \approx \frac{\chi^2}{2\eta} = \frac{(IP + EA)^2}{4(IP - EA)} \tag{S6}$$

This descriptor was introduced by Robert Parr and coworkers [53]. It measures the energy change of an electrophilic reagent (acceptor) when it is saturated with electrons provided by another reagent (donor). Higher values of ω characterize a strong electrophile, whereas a strong nucleophile is described by lower values of ω.

Electro-donating and electro-accepting power

A radical scavenger (RS) can act in two ways: either by donating electrons to a radical (R) or by accepting electrons from it

$$RS + R \rightarrow RS^{+} + R^{-} \qquad RS + R \rightarrow RS^{-} + R^{+}$$

Following this fact, Gázquez et al. [54] introduced new descriptors characterizing the electro-donating ω^- and electro-accepting ω^+ power of RS, defined in the following manner

$$\omega^- = \frac{(3IP + EA)^2}{16(IP - EA)} \qquad \omega^+ = \frac{(IP + 3EA)^2}{16(IP - EA)} \tag{S7}$$

To characterize the relative electro-accepting (donating) power of an arbitrary radical scavenger X, Martinez [55] introduced the acceptance Ra and donation Rd indexes

$$Ra = \frac{\omega_X^+}{\omega_F^+} \qquad Rd = \frac{\omega_X^-}{\omega_{Na}^-} \tag{S8}$$

defined by IP and EA for the F and Na atoms. They represent a good electron acceptor (F) and a good donor (Na), respectively. In the calculations, the well-known experimental values [55] for F: IP = 17.42282, EA = 3.4011898 [eV], and for Na: IP = 5.1391, EA = 0.547926 [eV] are employed. The descriptors Ra and Rd are useful for classifying any compound X in terms of its electron donating (accepting) capacity. Hence, Ra and Rd can be applied to characterize the antiradical capacity of X in terms of its electro-donating (accepting) power with respect to the F and Na atoms, taken as the reference points [55].

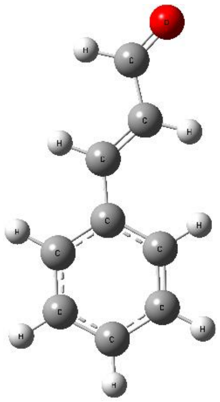

CI I E =–423.178463

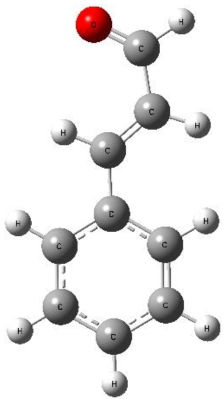

CI II E =–423.174149

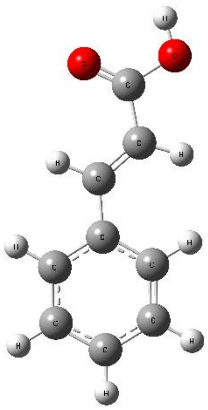

CIO I E =–498.470043

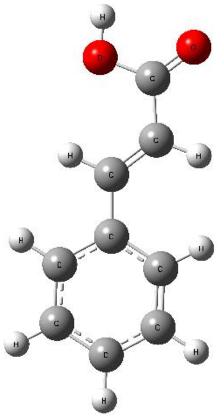

CIO II E =–498.469515

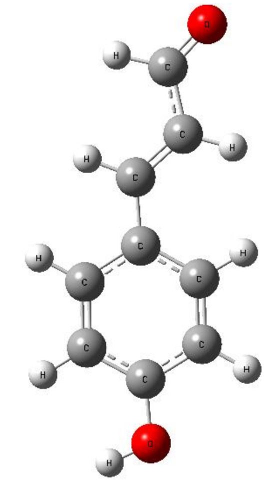

CO I E =–498.445602

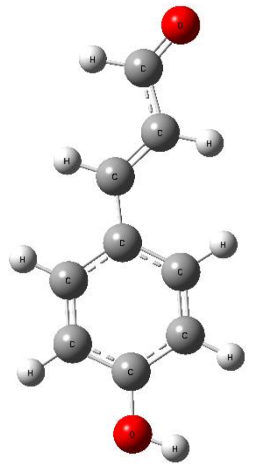

CO II E =–498.445532

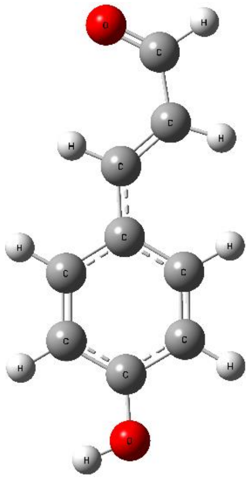

CO III E =–498.441293

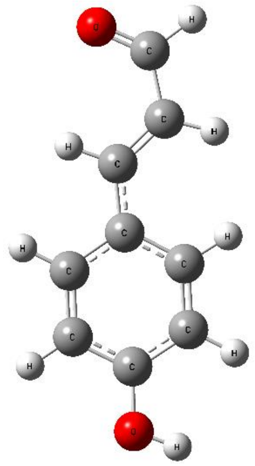

CO IV E =–498.441236

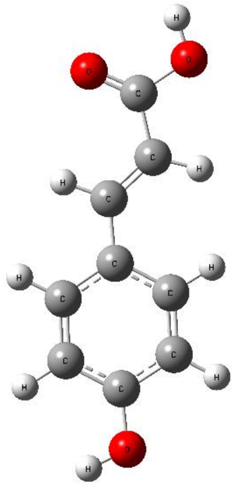

COO I E =–573.736842

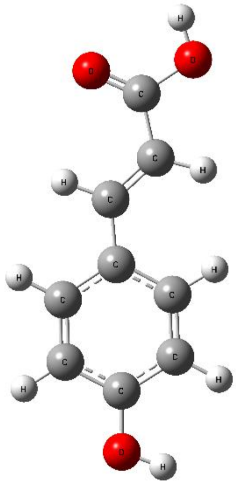

COO II E =–573.736766

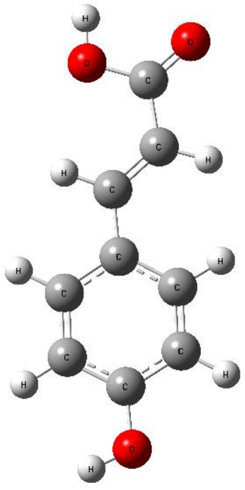

COO III E =–573.736286

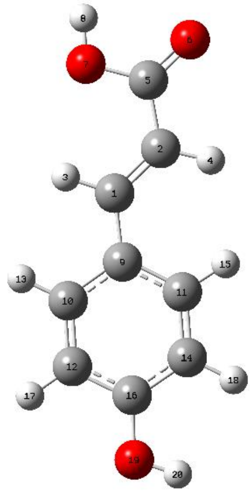

COO IV E =–573.736215

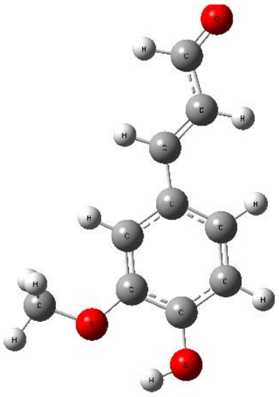

FE I E =–613.024053

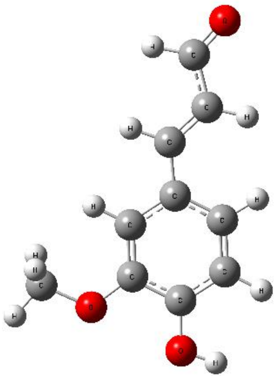

FE II E =–613.020416

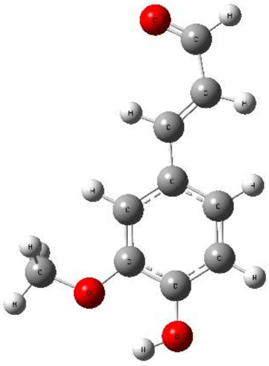

FE III E =–613.019796

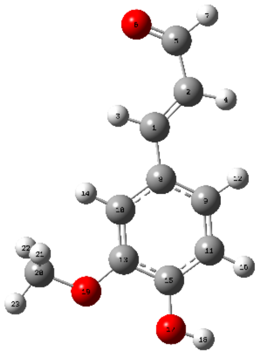

FE IV E =–613.016109

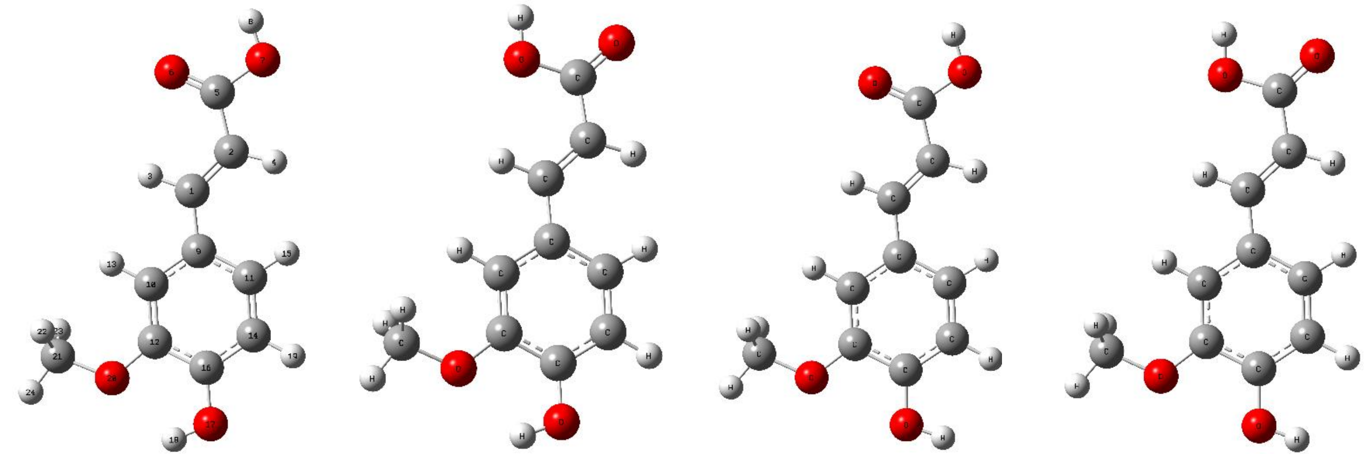

FEO I E =–688.315241 **FEO II E =–688.314691** **FEO III E =–688.311591** **FEO IV E =–688.311043**

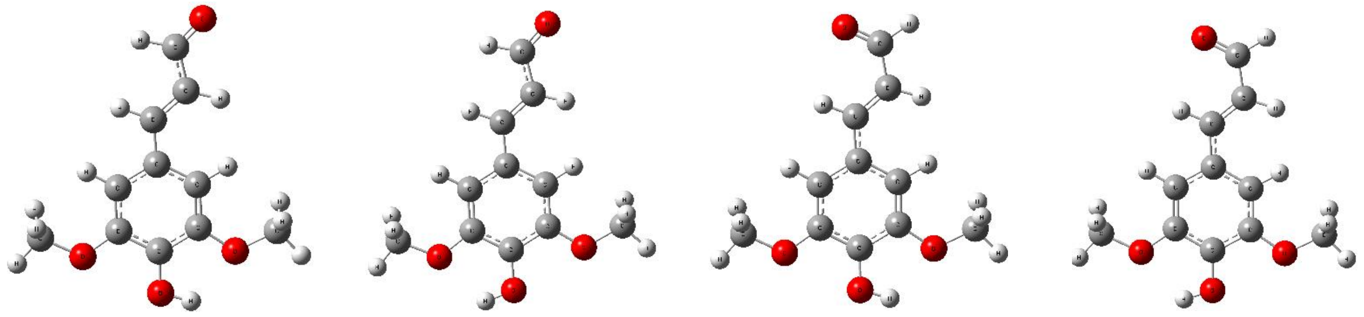

SI I E =–727.599247 **SI II E =–727.599171** **SI III E =–727.595000** **SI IV E =–727.594951**

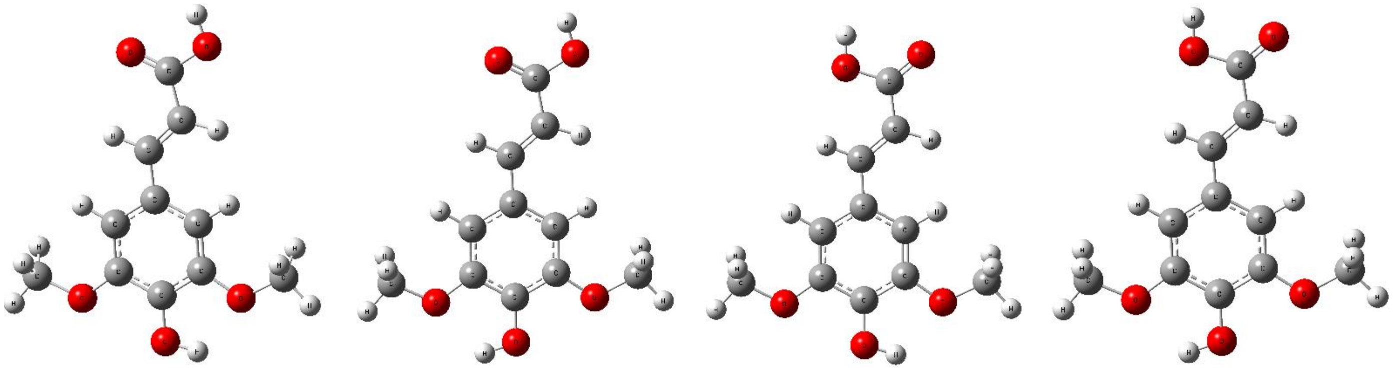

SIO I E = –802.890413 **SIO II E = –802.890357** **SIO III E = –802.889863** **SIO IV E = –802.889808**

Figure S1. The optimized geometries and energies [Ha] of the trans-phenylpropanoids under consideration evaluated by the DFT method at the B3LYP/QZVP theory level, using the C-PCM solvation model and the water medium. The energies of the planar conformers are presented and ranked in ascending order from I to IV. The non-planar conformers, which arise due to the up-down orientation of the methoxy group(s), exhibit higher energies compared to the planar structures displayed.

**HOMO**	$\xrightarrow{\Delta E}$	**LUMO**
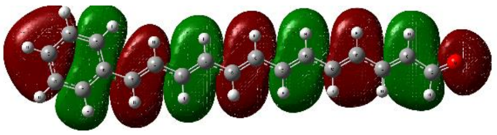		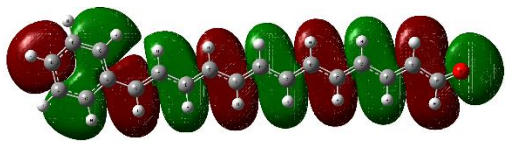
CIP6 E = -5.4083	2.4047	CIP6 E = -3.0036
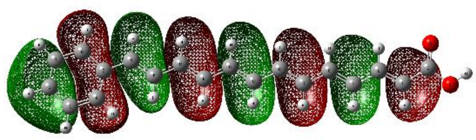		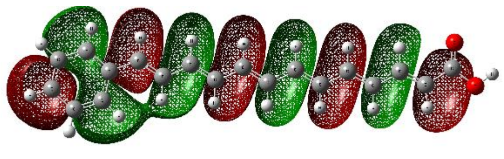
CIOPO6 E = -5.3802	2.9021	CIOPO6 E = -2.9021

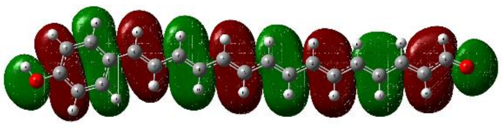
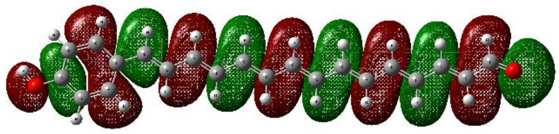

COP7 E = -5.1797 | 2.1717 | COP7 E = -3.0070

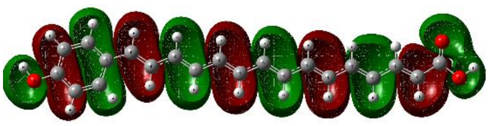
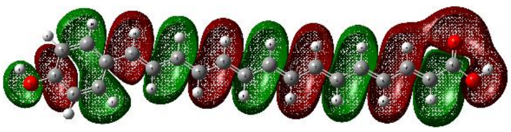

COOPO7 E = -5.1571 | 2.2458 | COOPO7 E = -2.9113

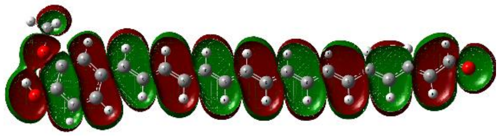
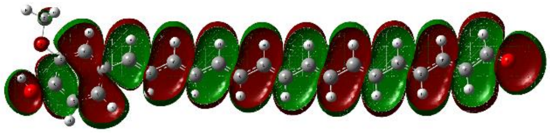

FEP8 E = -5.0738 | 2.0227 | FEP8 E = -3.0469

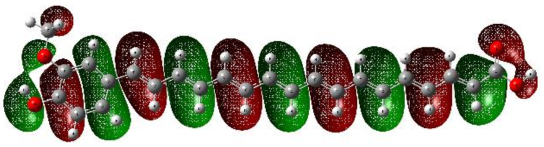
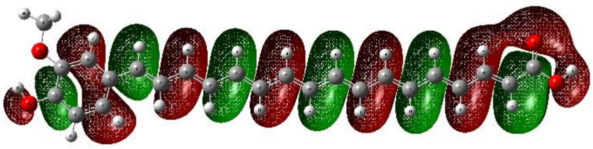

FEOPO8 E = -5.0542 | 2.0972 | FEOPO8 E = -2.9571

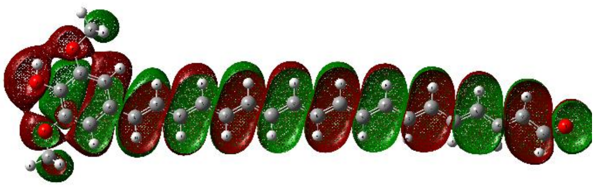
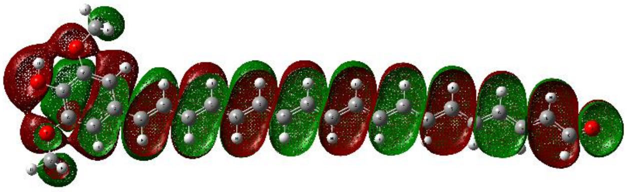

SIP9 E = -4.9960 | 1.9170 | SIP9 E = -3.0790

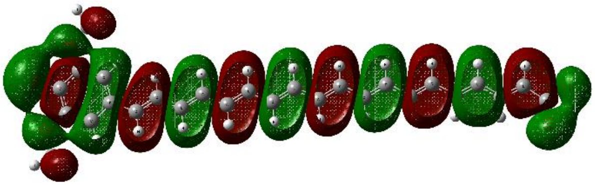
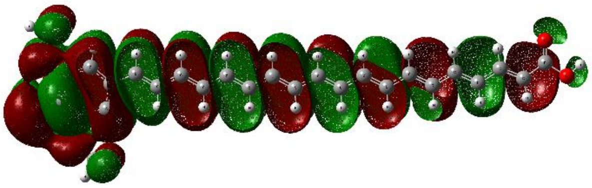

SIOPO9 E = -4.9786 | 1.9840 | SIOPO9 E = -2.9946

Figure S2. The HOMO–LUMO energies [eV] and frontier orbitals of the modeled compounds in water medium, calculated using the DFT B3LYP/QZVP level of the theory and the C-PCM solvation model. The calculations for the energy gap ΔE and molecular orbitals (isovalue 0.02) were accomplished using method I.

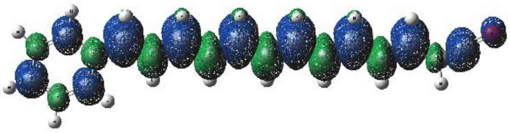
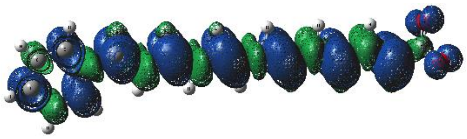

CIP6 R1 | CIOPO6 R1

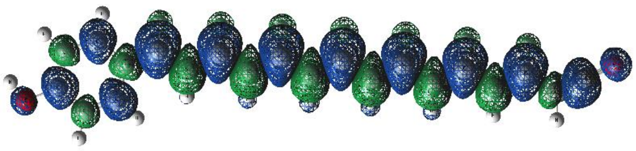
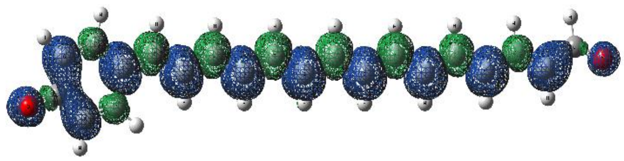

COP7 R1

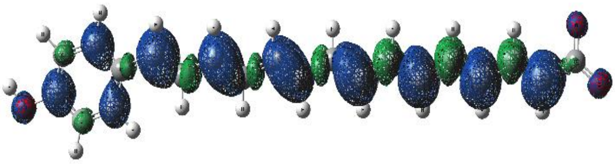

COP7 R2

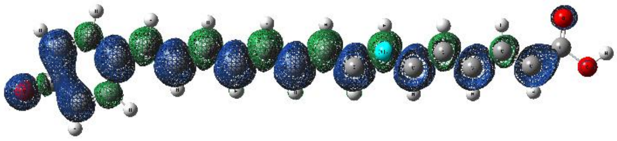

COOPO7 R1

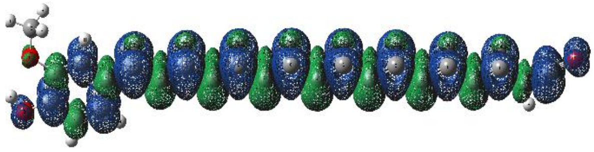

COOPO7 R2

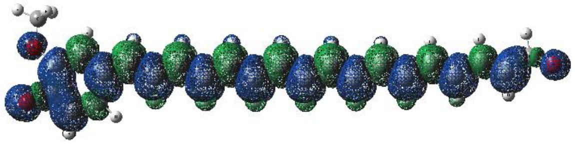

FEP8 R1

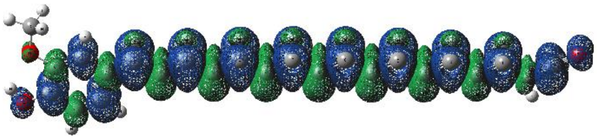

FEP8 R2

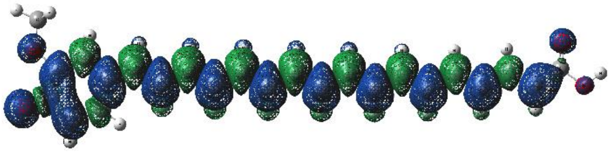

FEOPO8 R1

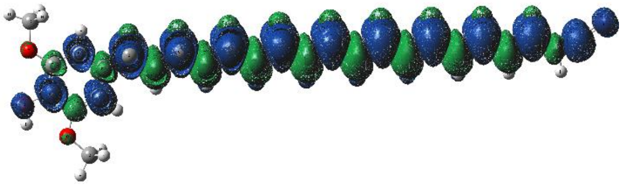

FEOPO8 R2

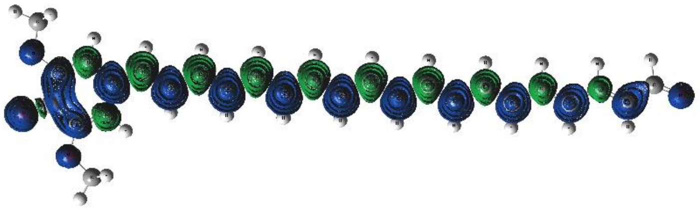

SIP9 R1

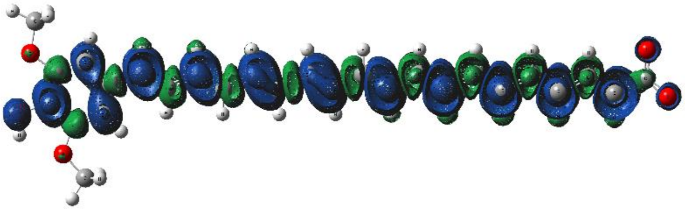

SIP9 R2

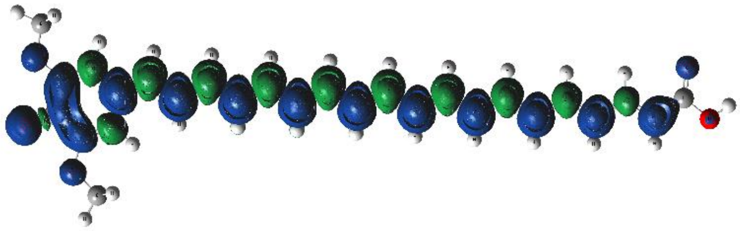

SIOPO9 R1

SIOPO9 R2

Figure S3. The spin density (isovalue 0.0004) for the radical forms of the modeled compounds calculated in water medium, using the DFT B3LYP/QZVP level of the theory and the C-PCM solvation model. The radicals are generated by the dehydrogenation of aldehyde O=C–H or carboxylic O=CO–H moieties (R1) and CO–H phenolic group (R2), respectively.

Table S1. The global chemical activity descriptors [eV] of cinnamaldehyde (CI), cinnamic acid (CIO), tetradecahexenal (P6), tetradecahexenoic acid (PO6) and their combinations (CIP6, CIOPO6) presented in Figures 2 and 3. The calculations were performed in the water medium at the B3LYP/QZVP theory level, using the C-PCM solvation model and methods I, II, and III. The energy values utilized in the calculations for methods II and III are provided.

**Descriptor**	**CI**	**CIP6**	**P6**	**CIO**	**CIOPO6**	**PO6**
			Method I			
EA	2.4980	3.0036	2.9029	2.3160	2.9021	2.7856
IP	6.8586	5.4083	5.4771	6.8175	5.3802	5.4453
ΔE	4.3606	2.4047	2.5742	4.5016	2.4781	2.6596
η	2.1803	1.2023	1.2871	2.2508	1.2391	1.3298

S	0.2293	0.4159	0.3885	0.2221	0.4035	0.3760
$\chi = -\mu$	4.6783	4.2059	4.1900	4.5668	4.1412	4.1155
ω	5.0189	7.3564	6.8201	4.6329	6.9202	6.3681
$\omega+$	2.9525	5.4038	4.8859	2.6308	5.0045	4.4766
$\omega-$	7.6308	9.6097	9.0760	7.1976	9.1457	8.5921
Ra [b]	0.8679	1.5884	1.4362	0.7733	1.4711	1.3159
Rd [b]]	2.1992	2.7695	2.6157	2.0743	2.6358	2.4762
			Method II			
$E(R-H)$[a]	-423.1785	-810.3946	-618.5704	-498.4700	-885.6857	-693.8616
$E^0(R-H^{\bullet+})$[a]	-422.9311	-810.2023	-618.3755	-498.2230	-885.4943	-693.6677
$E^0(R-H^{\bullet-})$[a]	-423.2757	-810.5112	-618.6835	-498.5608	-885.7986	-693.9703
EA	2.6449	3.1726	3.0756	2.4697	3.0706	2.9580
IP	6.7322	5.2335	5.3066	6.7226	5.2070	5.2765
ΔE	4.0873	2.0610	2.2310	4.2529	2.1363	2.3185
η	2.0437	1.0305	1.1155	2.1265	1.0681	1.1593
S	0.2447	0.4852	0.4482	0.2351	0.4681	0.4313
$\chi = -\mu$	4.6886	4.2030	4.1911	4.5962	4.1388	4.1172
ω	5.3783	8.5715	7.8734	4.9671	8.0183	7.3113
$\omega+$	3.2895	6.5988	5.9173	2.9348	6.0824	5.3976
$\omega-$	7.9780	10.8018	10.1084	7.5309	10.2212	9.5149
Ra [b]	0.9669	1.9397	1.7394	0.8627	1.7879	1.5866
Rd [b]	2.2993	3.1131	2.9132	2.1704	2.9457	2.7422
			Method III			
$E(R-H^{\bullet+})$[a]	-422.9359	-810.2069	-618.3803	-498.2292	-885.4991	-693.6727
$E(R-H^{\bullet-})$[a]	-423.2837	-810.5180	-618.6907	-498.5700	-885.8058	-693.9780
EA	2.8649	3.3589	3.2696	2.7206	3.2683	3.1694
IP	6.6008	5.1087	5.1751	6.5530	5.0782	5.1402
ΔE	3.7359	1.7499	1.9055	3.8324	1.8099	1.9708
η	1.8680	0.8749	0.9528	1.9162	0.9049	0.9854
S	0.2677	0.5715	0.5248	0.2609	0.5525	0.5074
$\chi = -\mu$	4.7328	4.2338	4.2223	4.6368	4.1733	4.1548
ω	5.9958	10.2436	9.3560	5.6101	9.6228	8.7588
$\omega+$	3.8628	8.2361	7.3640	3.5312	7.6493	6.8046
$\omega-$	8.5957	12.4699	11.5863	8.1680	11.8226	10.9594
Ra []b	1.1355	2.4210	2.1646	1.0380	2.2485	2.0002
Rd [b]	2.4773	3.5938	3.3392	2.3540	3.4073	3.1585

[a] In Hartree [Ha] unit. [b] Dimensionless parameter.

Table S2. The global chemical activity descriptors [eV] of coumaric aldehyde (CO), coumaric acid (COO), hexadecaheptenal (P7), hexadecahepteoic acid (PO7), and their combinations (COP7, COOPO7) presented in Figures 2 and 3. The calculations were performed in the water medium at the B3LYP/QZVP theory level, using C-PCM solvation model and methods I, II, and III. The energy values utilized in the calculations for methods II and III are provided.

**Descriptor**	**CO**	**COP7**	**P7**	**COO**	**COOPO7**	**PO7**
			Method I			
EA	2.3372	3.0079	2.9633	2.1554	2.9113	2.8572
IP	6.3800	5.1797	5.3359	6.3413	5.1571	5.3073

ΔE	4.0428	2.1717	2.3726	4.1859	2.2458	2.4501
η	2.0214	1.0859	1.1863	2.0930	1.1229	1.2251
S	0.2474	0.4605	0.4215	0.2389	0.4453	0.4081
$\chi = -\mu$	4.3586	4.0938	4.1496	4.2484	4.0342	4.0823
ω	4.6990	7.7170	7.2576	4.3118	7.2470	6.8016
ω+	2.7724	5.8058	5.3311	2.4492	5.3702	4.9136
ω−	7.1310	9.8996	9.4807	6.6976	9.4045	8.9959
Ra [b]	0.8149	1.7066	1.5671	0.7199	1.5786	1.4444
Rd [b]	2.0552	2.8531	2.7323	1.9302	2.7104	2.5926
Method II						
E(R—H)[a]	-498.4456	-963.1035	-696.0137	-573.7368	-1038.3945	-771.3048
$E^0(R{-}H^{\bullet+})$[a]	-498.2153	-962.9195	-695.8240	-573.5080	-1038.2076	-771.1160
$E^0(R{-}H^{\bullet-})$[a]	-498.5370	-963.2204	-696.1290	-573.8218	-1038.5148	-771.4161
EA	2.4880	3.1804	3.1368	2.3130	3.2740	3.0300
IP	6.2668	5.0083	5.1620	6.2280	5.0853	5.1354
ΔE	3.7788	1.8278	2.0252	3.9150	1.8114	2.1054
η	1.8894	0.9139	1.0126	1.9575	0.9057	1.0527
S	0.2646	0.5471	0.4938	0.2554	0.5521	0.4750
$\chi = -\mu$	4.3774	4.0943	4.1494	4.2705	4.1796	4.0827
ω	5.0708	9.1714	8.5015	4.6584	9.6444	7.9170
ω+	3.1183	7.2385	6.5534	2.7678	7.6678	6.0072
ω−	7.4957	11.3328	10.7027	7.0383	11.8474	10.0899
Ra [b]	0.9166	2.1277	1.9263	0.8136	2.2539	1.7658
Rd [b]	2.1603	3.2661	3.0845	2.0285	3.4144	2.9079
Method III						
$E(R{-}H^{\bullet+})$[a]	-498.2210	-962.9244	-695.8288	-573.5140	-1038.2163	-771.1209
$E(R{-}H^{\bullet-})$[a]	-498.5450	-963.2270	-696.1359	-573.8310	-1038.5148	-771.4236
EA	2.7046	3.3610	3.3259	2.5609	3.2740	3.2328
IP	6.1129	4.8741	5.0324	6.0647	4.8493	5.0018
ΔE	3.4083	1.5130	1.7064	3.5038	1.5753	1.7689
η	1.7041	0.7565	0.8532	1.7519	0.7877	0.8845
S	0.2934	0.6609	0.5860	0.2854	0.6348	0.5653
$\chi = -\mu$	4.4087	4.1176	4.1792	4.3128	4.0616	4.1173
ω	5.7028	11.2055	10.2350	5.3085	10.4719	9.5832
ω+	3.7114	9.2413	8.2521	3.3711	8.5396	7.6351
ω−	8.1202	12.4699	12.4313	7.6839	12.6012	11.7524
Ra [b]	1.0910	2.7165	2.4257	0.9909	2.5102	2.2443
Rd [b]	2.3402	3.8500	3.5827	2.2145	3.6317	3.3870

[a] In Hartree [Ha] unit. [b] Dimensionless parameter.

Table S3. The global chemical activity descriptors [eV] of ferulic aldehyde (FE), ferulic acid (FEO), octadecaoctenal (P8), octadecaoctenaenoic acid (PO8) , and their combinations (FEP8, FEOPO8) presented in Figures 2 and 3. The calculations were performed in the water medium at the B3LYP/QZVP theory level, using the C-PCM solvation model and methods I, II, and III. The energy values utilized in the calculations for methods II and III are provided.

**Descriptor**	**FE**	**FEP8**	**P8**	**FEO**	**FEOPO8**	**PO8**

Method I						
EA	2.3405	3.0469	3.0112	2.1576	2.9571	2.9105
IP	6.1217	5.0738	5.2235	6.0804	5.0542	5.1990
ΔE	3.7813	2.0270	2.2123	3.9228	2.0972	2.2885
η	1.8906	1.0135	1.1061	1.9614	1.0486	1.1442
S	0.2645	0.4933	0.4520	0.2549	0.4768	0.4370
χ = −μ	4.2311	4.0603	4.1174	4.1190	4.0057	4.0548
ω	4.7344	8.1335	7.6629	4.3250	7.6509	7.1843
ω+	2.8552	6.2300	5.7425	2.5107	5.7791	5.3000
ω−	7.0863	10.2903	9.8599	6.6297	9.7848	9.3547
Ra [b]	0.8393	1.8313	1.6880	0.7380	1.6988	1.5579
Rd [b]	2.0423	2.9657	2.8416	1.9107	2.8200	2.6960
Method II						
E(R—H)[a]	-613.0241	-1155.1252	-773.4570	-688.3152	-1230.4162	-848.7480
E^0(R—$H^{\bullet+}$)[a]	-612.8032	-1154.9450	-773.2714	-688.0958	-1230.2366	-848.5633
E^0(R—$H^{\bullet-}$)[a]	-613.1156	-1155.2435	-773.5740	-688.4002	-1230.5311	-848.8613
EA	2.4909	3.2185	3.1842	2.3131	3.1274	3.0854
IP	6.0087	4.9046	5.0483	5.9720	4.8864	5.0252
ΔE	3.5177	1.6861	1.8642	3.6589	1.7590	1.9398
η	1.7589	0.8430	0.9321	1.8294	0.8794	0.9699
S	0.2843	0.5931	0.5364	0.2733	0.5685	0.5155
χ = −μ	4.2498	4.0615	4.1163	4.1425	4.0069	4.0523
ω	5.1342	9.7834	9.0890	4.6901	9.1274	8.4782
ω+	3.2292	7.8580	7.1474	2.8476	7.2339	6.5718
ω−	7.4790	11.9195	11.2637	6.9901	11.2408	10.6271
Ra [b]	0.9492	2.3099	2.1010	0.8370	2.1264	1.9318
Rd [b]	2.1554	3.4352	3.2462	2.0145	3.2396	3.0627
Method III						
E(R—$H^{\bullet+}$)[a]	-612.8096	-1154.9499	-773.2761	-688.1024	-1230.2416	-848.5682
E(R—$H^{\bullet-}$)[a]	-613.1236	-1155.2501	-773.5808	-688.4095	-1230.5380	-848.8686
EA	2.7083	3.3975	3.3697	2.5658	3.3150	3.2822
IP	5.8353	4.7719	4.9200	5.7912	4.7508	4.8931
ΔE	3.1270	1.3744	1.5504	3.2254	1.4357	1.6109
η	1.5635	0.6872	0.7752	1.6127	0.7179	0.8055
S	0.3198	0.7276	0.6450	0.3100	0.6965	0.6208
χ = −μ	4.2718	4.0847	4.1449	4.1785	4.0329	4.0876
ω	5.8357	12.1399	11.0810	5.4133	11.3282	10.3720
ω+	3.8953	10.1835	9.1054	3.5256	9.4015	8.4289
ω−	8.1670	14.2682	13.2503	7.7041	13.4344	12.5165
Ra []b	1.1450	2.9934	2.6765	1.0363	2.7635	2.4776
Rd [b]	2.3537	4.0847	3.8187	2.2203	3.8718	3.6073

[a] In Hartree [Ha] unit. [b] Dimensionless parameter.

Table S4. The global chemical activity descriptors [eV] of sinapic aldehyde (SI), sinapic acid (SIO), eicosanonenal (P9), eicosanonenaoic acid (PO9), and their combinations SIP9 and SIOPO9 presented in Figures 2 and 3. The calculations were performed in the water medium at the B3LYP/QZVP theory level, using the C-PCM solvation model and methods I, II, and III. The energy values utilized in the calculations for methods II and III are provided.

Descriptor	SI	SIP9	P9	SIO	SIOPO9	PO9
			Method I			
EA	2.3434	3.0790	3.0488	2.1617	2.9946	2.9581
IP	6.0069	4.9960	5.1329	5.9677	4.9786	5.1108
ΔE	3.6635	1.9170	2.0841	3.8061	1.9840	2.1527
η	1.8317	0.9585	1.0421	1.9030	0.9920	1.0763
S	0.2730	0.5216	0.4798	0.2627	0.5040	0.4645
χ = −μ	4.1752	4.0375	4.0908	4.0647	3.9866	4.0345
ω	4.7584	8.5034	8.0297	4.3409	8.0107	7.5613
ω+	2.8997	6.6044	6.1145	2.5464	6.1414	5.6786
ω−	7.0749	10.6419	10.2054	6.6111	10.1280	9.7131
Ra [b]	0.8524	1.9414	1.7974	0.7485	1.8052	1.6692
Rd [b]	2.0390	3.0670	2.9412	1.9053	2.9189	2.7993
			Method II			
E(R—H)[a]	-727.5992	-1347.1436	-850.9002	-802.8904	-1422.4346	-926.1912
E^0(R—$H^{\bullet+}$)[a]	-727.3829	-1346.9662	-850.7180	-802.6754	-1422.2577	-926.0098
E^0(R—$H^{\bullet-}$)[a]	--727.6909	-1347.2630	-851.0186	-802.9756	-1422.5509	-926.3062
EA	2.4926	3.2487	3.2207	2.3169	3.1630	3.1290
IP	5.8867	4.8292	4.9578	5.8507	4.8133	4.9372
ΔE	3.3940	1.5804	1.7371	3.5338	1.6502	1.8082
η	1.6970	0.7902	0.8685	1.7669	0.8251	0.9041
S	0.2946	0.6327	0.5757	0.2830	0.6060	0.5530
χ = −μ	4.1897	4.0389	4.0892	4.0838	3.9881	4.0331
ω	5.1718	10.3218	9.6264	4.7194	9.6382	8.9956
ω+	3.2891	8.4011	7.6904	2.8983	7.7473	7.0921
ω−	7.4788	12.4401	11.7796	6.9821	11.7354	11.1252
Ra [b]	0.9668	2.4695	2.2606	0.8520	2.2773	2.0847
Rd [b]	2.1554	3.5852	3.3949	2.0122	3.3821	3.2063
			Method III			
E(R—$H^{\bullet+}$)[a]	-727.3895	-1346.9674	-850.7227	-802.6822	-1422.2627	-926.0146
E(R—$H^{\bullet-}$)[a]	-727.6989	-1347.2696	-851.0253	-802.9849	-1422.5577	-926.3133
EA	2.7119	3.4268	3.4043	2.5700	3.3482	3.3214
IP	5.7066	4.7949	4.8297	5.6642	4.6779	4.8058
ΔE	2.9947	1.3681	1.4254	3.0942	1.3297	1.4844
η	1.4973	0.6841	0.7127	1.5471	0.6649	0.7422
S	0.3339	0.7309	0.7016	0.3232	0.7520	0.6737
χ = −μ	4.2092	4.1109	4.1170	4.1171	4.0131	4.0636
ω	5.9163	12.3524	11.8914	5.4781	12.1112	11.1241
ω+	3.9988	10.3825	9.9220	3.6129	10.1878	9.1850
ω−	8.2080	14.4934	14.0390	7.7300	14.2008	13.2486
Ra [b]	1.1754	3.0519	2.9166	1.0620	2.9947	2.6999
Rd [b]	2.3656	4.1770	4.0460	2.2278	4.0927	3.8183

[a] In Hartree [Ha] unit. [b] Dimensionless parameter.
